# Electron Transport Lipids Fold Within Membrane-Like Interfaces

**DOI:** 10.3389/fchem.2022.827530

**Published:** 2022-03-08

**Authors:** Margaret M. Braasch-Turi, Jordan T. Koehn, Kateryna Kostenkova, Cameron Van Cleave, Jacob W. Ives, Heide A. Murakami, Dean C. Crick, Debbie C. Crans

**Affiliations:** ^1^ Chemistry Department, Colorado State University, Fort Collins, CO, United States; ^2^ Cell and Molecular Biology Program, Colorado State University, Fort Collins, CO, United States; ^3^ Microbiology, Immunology, and Pathology Department, Colorado State University, Fort Collins, CO, United States

**Keywords:** lipoquinone, ubiquinone, menaquinone, folded conformation, 2D NMR, electron transport, membrane interface, Langmuir monolayer

## Abstract

Lipoquinones, such as ubiquinones (UQ) and menaquinones (MK), function as essential lipid components of the electron transport system (ETS) by shuttling electrons and protons to facilitate the production of ATP in eukaryotes and prokaryotes. Lipoquinone function in membrane systems has been widely studied, but the exact location and conformation within membranes remains controversial. Lipoquinones, such as Coenzyme Q (UQ-10), are generally depicted simply as “Q” in life science diagrams or in extended conformations in primary literature even though specific conformations are important for function in the ETS. In this study, our goal was to determine the location, orientation, and conformation of UQ-2, a truncated analog of UQ-10, in model membrane systems and to compare our results to previously studied MK-2. Herein, we first carried out a six-step synthesis to yield UQ-2 and then demonstrated that UQ-2 adopts a folded conformation in organic solvents using ^1^H-^1^H 2D NOESY and ROESY NMR spectroscopic studies. Similarly, using ^1^H-^1^H 2D NOESY NMR spectroscopic studies, UQ-2 was found to adopt a folded, U-shaped conformation within the interface of an AOT reverse micelle model membrane system. UQ-2 was located slightly closer to the surfactant-water interface compared to the more hydrophobic MK-2. In addition, Langmuir monolayer studies determined UQ-2 resided within the monolayer water-phospholipid interface causing expansion, whereas MK-2 was more likely to be compressed out and reside within the phospholipid tails. All together these results support the model that lipoquinones fold regardless of the headgroup structure but that the polarity of the headgroup influences lipoquinone location within the membrane interface. These results have implications regarding the redox activity near the interface as quinone vs. quinol forms may facilitate locomotion of lipoquinones within the membrane. The location, orientation, and conformation of lipoquinones are critical for their function in generating cellular energy within membrane ETS, and the studies described herein shed light on the behavior of lipoquinones within membrane-like environments.

## 1 Introduction

**FIGURE 1:**
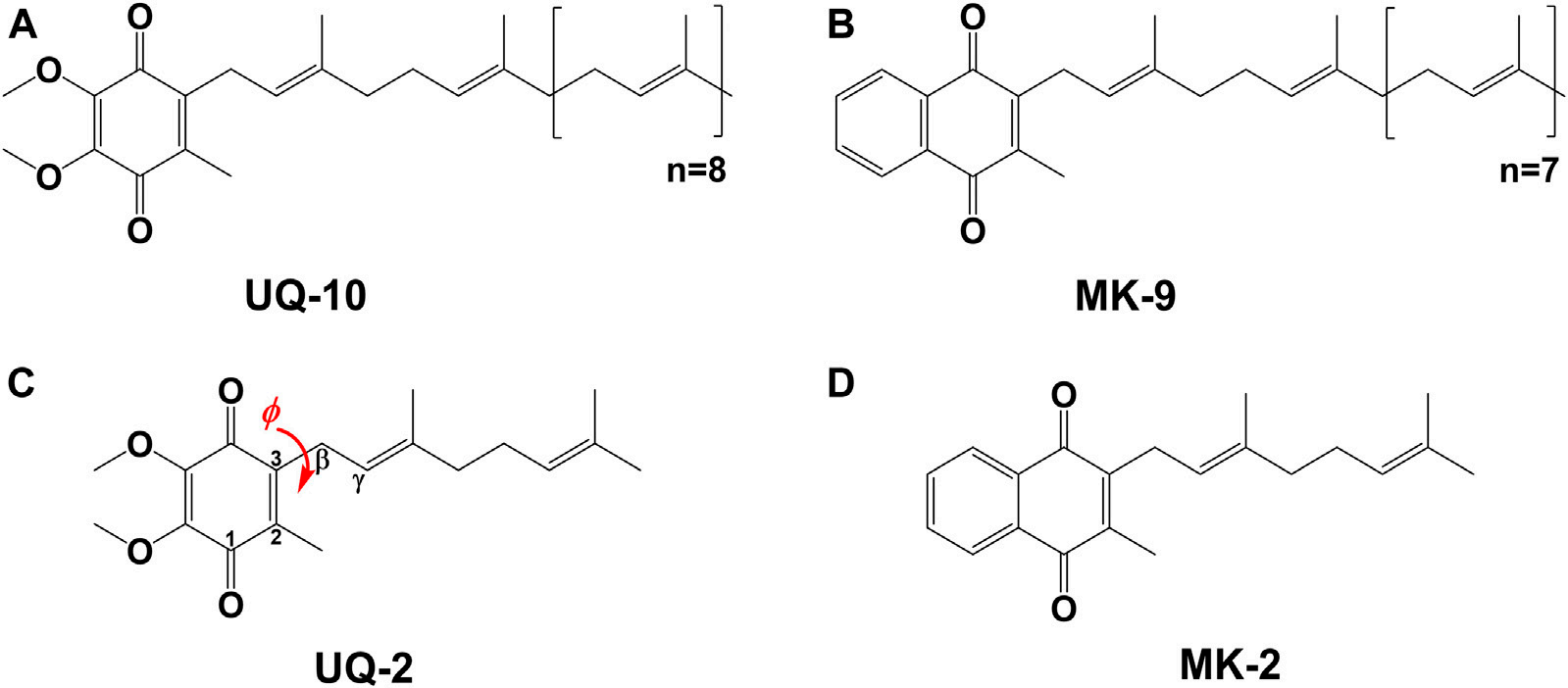
Schematic structures of native lipoquinones: **(A)** Ubiquinone-10 (UQ-10), and **(B)** Menaquinone-9 (MK-9). Schematic structures of truncated lipoquinone analogs: **(C)** Ubiquinone-2 (UQ-2) with the C2C3CβCγ bond indicated in red with dihedral angle, φ, and **(D)** Menaquinone-2 (MK-2).

Molecular conformations are paramount to the physical and chemical properties that dictate recognition and function of molecules within biological systems. The location and conformation of lipoquinones within biological membranes is not well understood and highly debated (Kingsley and Feigenson, 1981; Michaelis and Moore, 1985; Ulrich et al., 1985; Salgado et al., 1993; Soderhall and Laaksonen, 2001; Afri et al., 2004; Galassi and Arantes, 2015; Quirk et al., 2016; Koehn et al., 2018b; Koehn et al., 2019). Lipoquinones are hydrophobic membrane-bound molecules consisting of a redox-active quinone headgroup and an isoprenyl side chain. There are three major structural subgroups of lipoquinones which differ only in the structure of the headgroup. Ubiquinones (UQ), such as Ubiquinone-10 (UQ-10, Figure 1A), comprise of a benzoquinone ring with two methoxy substituents, plastoquinones (PQ) with a dimethylbenzoquinone, such as plastoquinone-9 (PQ-9), and menaquinones (MK), such as menaquinone-9 (MK-9, Figure 1B), contain a methylnaphthoquinone ring. Lipoquinones function as essential components of the respiratory electron transport system (ETS), where they shuttle electrons and protons between membrane-bound protein complexes, ultimately ending in the production of ATP (Nowicka and Kruk, 2010; Kawamukai, 2018). UQ and MK are involved in the ETS of oxidative phosphorylation in mammalian and bacterial cells, but PQ is involved in photosynthetic ETS in plants and photosynthetic bacteria (Kawamukai, 2018). For the purpose of this study, we will focus on UQ and MK. Even though the ETS is vital for life through the production of ATP, the role of lipoquinones is commonly distilled to an abbreviation within a diagram, such as “Q” for Coenzyme Q (UQ-10), the major electron transport agent in eukaryotes (Trumpower, 1981; Kawamukai, 2018), ignoring the conformation and location of these molecules as a whole. Similar to lipoquinones, polyprenyl compounds have been known to adopt preorganized, folded conformations presumably due to hydrophobic effect and π-π interactions (Woodward and Bloch, 1953; Murgolo et al., 1989). The synthesis of cholesterol relies on the preorganized conformation of squalene epoxide to produce a single stereochemical outcome out of 256 (2^8^) possible conformations (Woodward and Bloch, 1953). Moreover, dolichol-19 adopts a coiled conformation (Murgolo et al., 1989). A handful of computational studies have investigated the dihedral angle (φ) about the C2C3CβCγ bond (as shown in red in Figure 1C) in UQs (Nilsson et al., 2001b; Ceccarelli et al., 2003; Galassi and Arantes, 2015; Eddine et al., 2020), MKs (Eddine et al., 2020), and plastoquinones (Nilsson et al., 2001a; Jong et al., 2015; Eddine et al., 2020), which determined φ was ∼90°, 100°, and 90°, respectively. In this study we determined the location, orientation, and conformation of UQ-2 (Figure 1C), a truncated, representative analog for native UQ-10, using 1D and 2D NMR spectroscopic methods in organic solvents and in biological model membrane systems comprised of AOT reverse micelles (RM) (Van Horn et al., 2008). This analysis will allow us to compare the location and conformation of UQ-2 with MK-2 (Figure 1D) (Koehn et al., 2018b) in membrane-like environments to shed light on the controversies regarding the location and conformation of lipoquinones in cellular membranes.

**FIGURE 2:**
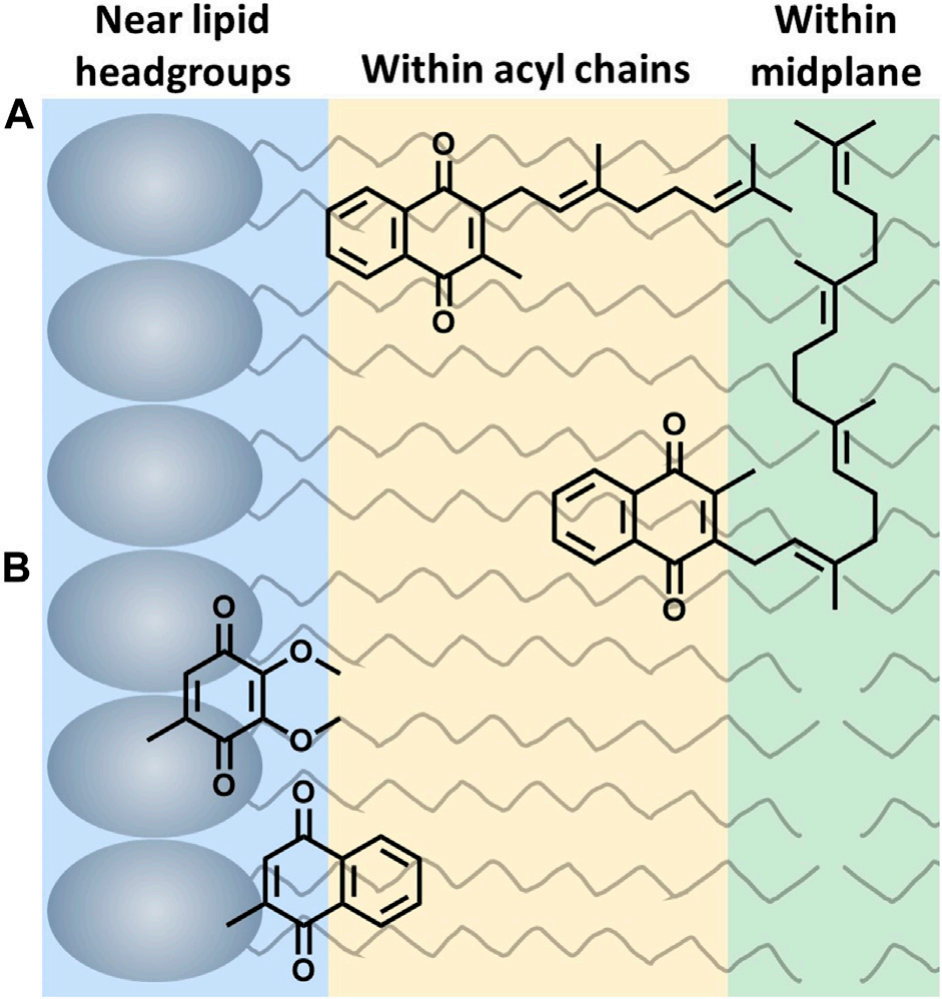
Three possible locations of lipoquinone headgroup: **(A)** Example of the influence of the isoprenyl side chain length on location of the quinone headgroup. MK-2 and MK-4 are shown as a 2D schematics. MK-2 has been determined to fold in membrane-like interfaces (Koehn et al., 2018b). **(B)** Relative placement based on headgroup polarity (side chain omitted for clarity) (Van Cleave et al., 2020; Van Cleave et al., 2021).

The location of UQ-10 within the membrane has been widely studied using experimental and computational methods, but it continues to be controversial. Briefly, there is no consensus regarding the location of UQ-10 with its locations spanning the entire width of the membrane bilayer leaflet. Out of these studies, three schools of thought have emerged; the quinone headgroup is located: 1) at or near the lipid headgroups (Kingsley and Feigenson, 1981; Stidham et al., 1984; Lenaz et al., 1992; Salgado et al., 1993; Galassi and Arantes, 2015; Gómez-Murcia et al., 2016; Kaurola et al., 2016; Quirk et al., 2016; Teixeira and Arantes, 2019), 2) within the acyl chains (Michaelis and Moore, 1985; Cornell et al., 1987; Chazotte et al., 1991; Salgado et al., 1993; Metz et al., 1995; Afri et al., 2004; Hauss et al., 2005), or 3) within the bilayer midplane (Ulrich et al., 1985; Ondarroa and Quinn, 1986; Soderhall and Laaksonen, 2001) (Figure 2). Even though the location of the headgroup is controversial, the field does seem to agree that at least part of the isoprenyl side chain is embedded within the bilayer midplane, and the headgroup is thought to extend into one of the membrane leaflets. This bend (∼90° turn) in the isoprenyl side chain allows UQ-10 to be accommodated within the bilayer, which addresses the fact that UQ-10 is roughly the same length as a typical phospholipid bilayer if UQ-10 were in fully-extended conformation (Trumpower, 1981).

Two hypotheses have been reported to explain the headgroup position within the membrane. First, the length of the isoprenyl side chain was reported to affect the position of the quinone headgroup by pulling the headgroup closer to the midplane as the side chain length increases. For example, the quinone headgroup of MK-4 would be found closer to the midplane than MK-2 (Figure 2A). On the other hand, the polarity of the headgroup is also thought to anchor its location within the membrane. Recent computational studies by Arantes and coworkers determined UQ associates with the lipid headgroups (Galassi and Arantes, 2015; Teixeira and Arantes, 2019). Together, we recently showed for the series of MK-1, MK-2, MK-3, and MK-4 that the location of the headgroup remained constant as the side chain length increased using both experimental and computational techniques (Van Cleave et al., 2020). As a consequence of the latter, one would anticipate a difference in the location of the more polar UQ headgroup compared to the more hydrophobic MK headgroup as illustrated in Figure 2B (Van Cleave et al., 2020). Lipoquinone headgroup is also linked to diffusive motion. “Swimming” lipoquinones are associated with the phospholipid headgroups, and “diving” are found near the midplane. A few computational and experimental studies determined UQ and various analogs are stabilized in the swimming position (Soderhall and Laaksonen, 2001; Hoyo et al., 2017). A recent computational study determined the lipoquinone position depends on the local protein content of the membrane (Singharoy et al., 2020). If the region is lipid-rich, swimming lipoquinone is the dominant species, and diving lipoquinones are the most common in the vicinity of protein complexes (Gupta et al., 2020; Singharoy et al., 2020).

Although lipoquinone conformation is likely to be critical for function and recognition, the topic of conformation of the UQ and MK headgroup relative to the isoprenyl side chain is curiously ignored in the literature aside from a handful of computational studies (Joela et al., 1997; Bernardo et al., 1998; Lenaz et al., 1999; Nilsson et al., 2001a; Nilsson et al., 2001b; Soderhall and Laaksonen, 2001; Ceccarelli et al., 2003; Tekin and Erkoc, 2010; Galassi and Arantes, 2015; Jong et al., 2015; Ismail et al., 2016; Kaurola et al., 2016; Eddine et al., 2020; Feng et al., 2021). Additionally, a few of the computational studies investigating the location of lipoquinones in the membrane contained figures suggesting φ was ∼90° (Joela et al., 1997; Bernardo et al., 1998; Lenaz et al., 1999; Soderhall and Laaksonen, 2001; Tekin and Erkoc, 2010; Ismail et al., 2016; Kaurola et al., 2016; Feng et al., 2021), leading to the expectation of a folded conformation. However, there was no discussion regarding the conformation of the headgroup relative to the isoprenyl side chain prior to our work in 2018 (Koehn et al., 2018b). The implications of conformation on lipoquinone locomotion were hypothesized by Joela and coworkers (Joela et al., 1997). Therein, they speculated the quinone headgroup is located close to the enzyme active site and moves between membrane and enzyme binding pocket by rotating about the C2C3CβCγ bond. They describe this limited movement with a stationary side chain and a mobile headgroup as if the “tail is wagging the dog.” The isoprenyl chain would serve to the anchor the quinone headgroup location. Since we previously found that the side chain did not dictate the headgroup location for MK-1 through MK-4, we hypothesized that the anchoring of the headgroup drives the extension of the side chain, and that the more polar UQ-2 headgroup will be closer to the interface than the more hydrophobic headgroup of MK-2. Hence, we carried out studies in which the location and conformation of UQ-2 were elucidated in environments that allow direct comparison to previously reported MK-2, which folds within model membrane interfaces (Koehn et al., 2018b). This study will illuminate how headgroup structure changes the position, orientation, and conformation, which are critical to recognition and function, of prominent lipoquinones within membrane-like environments.

## 2 Results and Discussion

### 2.1 Synthesis of UQ-2

**SCHEME 1:**
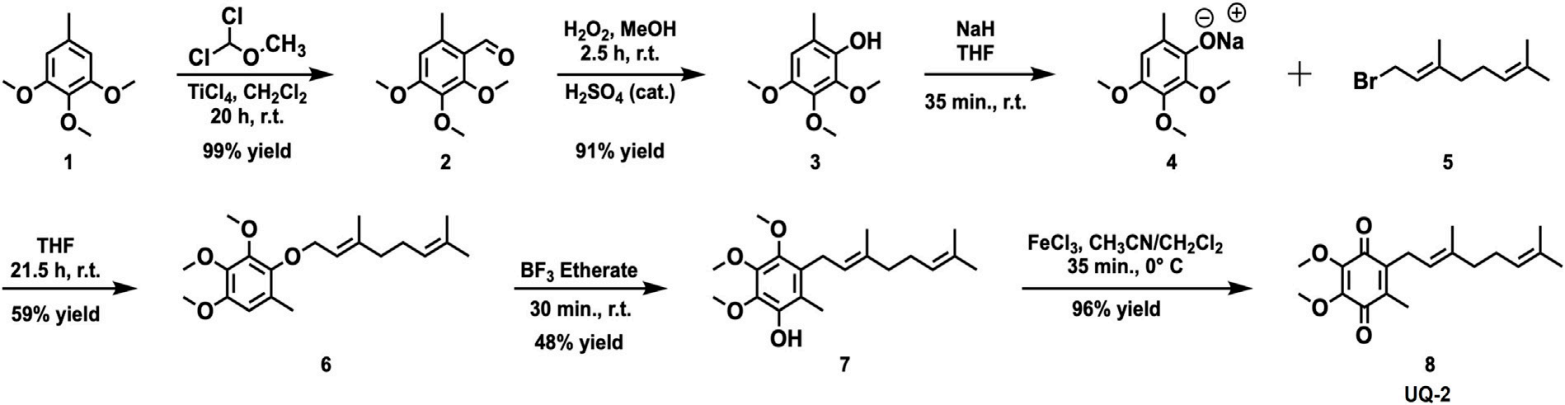
Synthetic scheme for the six-step synthesis to obtain UQ-2 **8** from 3,4,5-trimethoxytoluene **1** using modified protocols as reported (Matsumoto et al., 1984; Lu and Chen, 2004; Bovicelli et al., 2008).

UQ-2 **8** was prepared using a 6-step synthesis (Scheme 1) starting from commercially available 3,4,5-trimethoxytoluene **1**. The synthetic route has been reported in literature (Lu and Chen, 2004; Bovicelli et al., 2008); however, we scaled up the reactions and used modified procedures and conditions to overcome synthetic challenges encountered. The aldehyde **2** was prepared efficiently and was practically pure upon workup using a Rieche formylation reaction with TiCl_4_, which is the traditional Lewis acid catalyst for this reaction. Efforts to achieve this reaction using AlCl_3_ as the Lewis acid catalyst were low yielding and resulted in impure compound in our hands. An acid-catalyzed Dakin oxidation reaction was used to afford phenol **3** in excellent yield even after chromatographic purification (Matsumoto et al., 1984). The geranyl ether **6** was prepared via an S_N_2 reaction between phenolate **4** and geranyl bromide **5** in a modest yield. Efforts to achieve the allylic rearrangement to yield compound **7** close to yields reported in literature (Bovicelli et al., 2008) was met with limited success. A 48% yield was the highest yield we obtained compared to 73% in literature (Bovicelli et al., 2008). Attempts to improve this yield failed, and reactions times longer than 30 min decreased the yield and appeared to increase the amount of unknown side products. While compound **7** was efficiently oxidized to **8** using FeCl_3_∙6H_2_O in a mixture of dichloromethane and acetonitrile at 0°C, attempts to follow a published procedure (Bovicelli et al., 2008) using FeCl_3_ in a mixture of ethanol and H_2_O at ambient temperature yielded only starting material.

### 2.2 1D ^1^H NMR Spectroscopic Studies of UQ-2 in Organic Solvents

**FIGURE 3:**
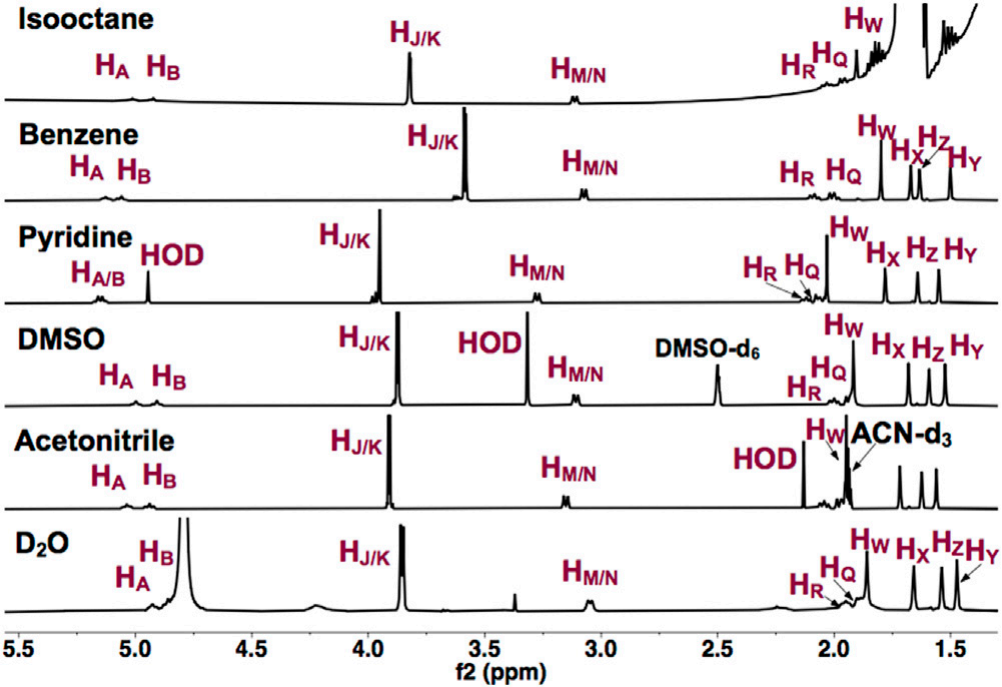
^1^H NMR (400 MHz) spectra of UQ-2 in hydrophobic (isooctane (2,2,4-trimethtylpentane), d_6_-benzene, and d_5_-pyridine) and hydrophilic (d_6_-DMSO, d_3_-acetonitrile, and D_2_O) solvents.

To establish that UQ conformation is sensitive to its surrounding environment, UQ-2 was first characterized using 1D ^1^H NMR spectroscopy. Figure 3 shows the 1D ^1^H NMR spectra of UQ-2 in isooctane (2,2,4-trimethylpentane), d_6_-benzene, d_5_-pyridine, d_6_-DMSO, d_3_-acetonitrile, and D_2_O. The observed chemical shifts of UQ-2 vary dramatically in the different solvents shown. Different spectroscopic trends are observed in the hydrophobic (isooctane, d_5_-pyridine, and d_6_-benzene) and the hydrophilic (d_3_-acetonitrile, d_6_-DMSO, and H_2_O/D_2_O) solvent environments. For example, the isoprenyl protons H_M_/H_N_ and methoxy protons H_J_/H_K_ are observed at significantly different chemical shifts between the two different classes of solvents. The observations could be described by conformational changes of UQ-2 in the various solvent environments investigated, alterations of the electronic state due to interaction with the solvent, or most likely, a combination of both. There were similarities and differences among the investigated solvents. For the hydrophobic solvents, protons H_M_/H_N_ have similar chemical shifts in isooctane and benzene, but they appear more downfield in pyridine, whereas they appear in similar chemical shifts in the hydrophilic solvents. The chemical shifts for H_J_ and H_K_ are in different locations in each solvent, which suggests the methoxy groups are changing environments in the different solvents (Nilsson et al., 2001b). The chemical shifts of the vinyl protons H_A_ and H_B_ are increasingly more downfield as the polarity of the hydrophobic solvents increases. In the hydrophilic solvents, H_A_ and H_B_ are found in similar chemical shifts aside from D_2_O where they appear slightly upfield and obscured by the HOD peak.

### 2.3 ^1^H-^1^H 2D NOESY and ^1^H-^1^H 2D ROESY NMR Spectroscopic Studies of UQ-2 in d_6_-DMSO, d_3_-Acetonitrile, d_6_-Benzene, and d_5_-Pyridine

**FIGURE 4:**
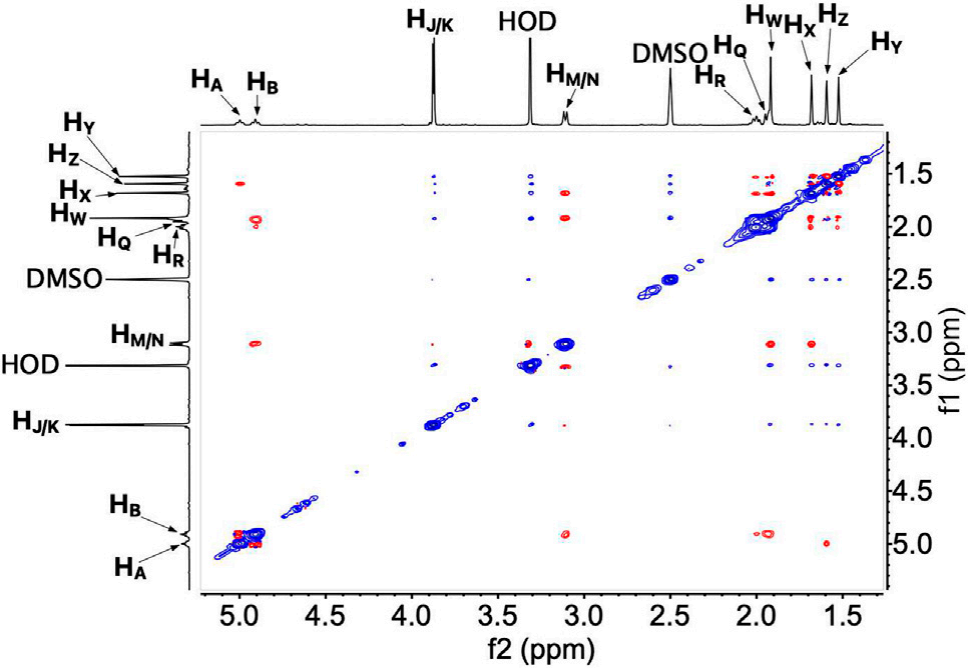
Full ^1^H-^1^H 2D NOESY spectrum of UQ-2 in DMSO.

**FIGURE 5:**
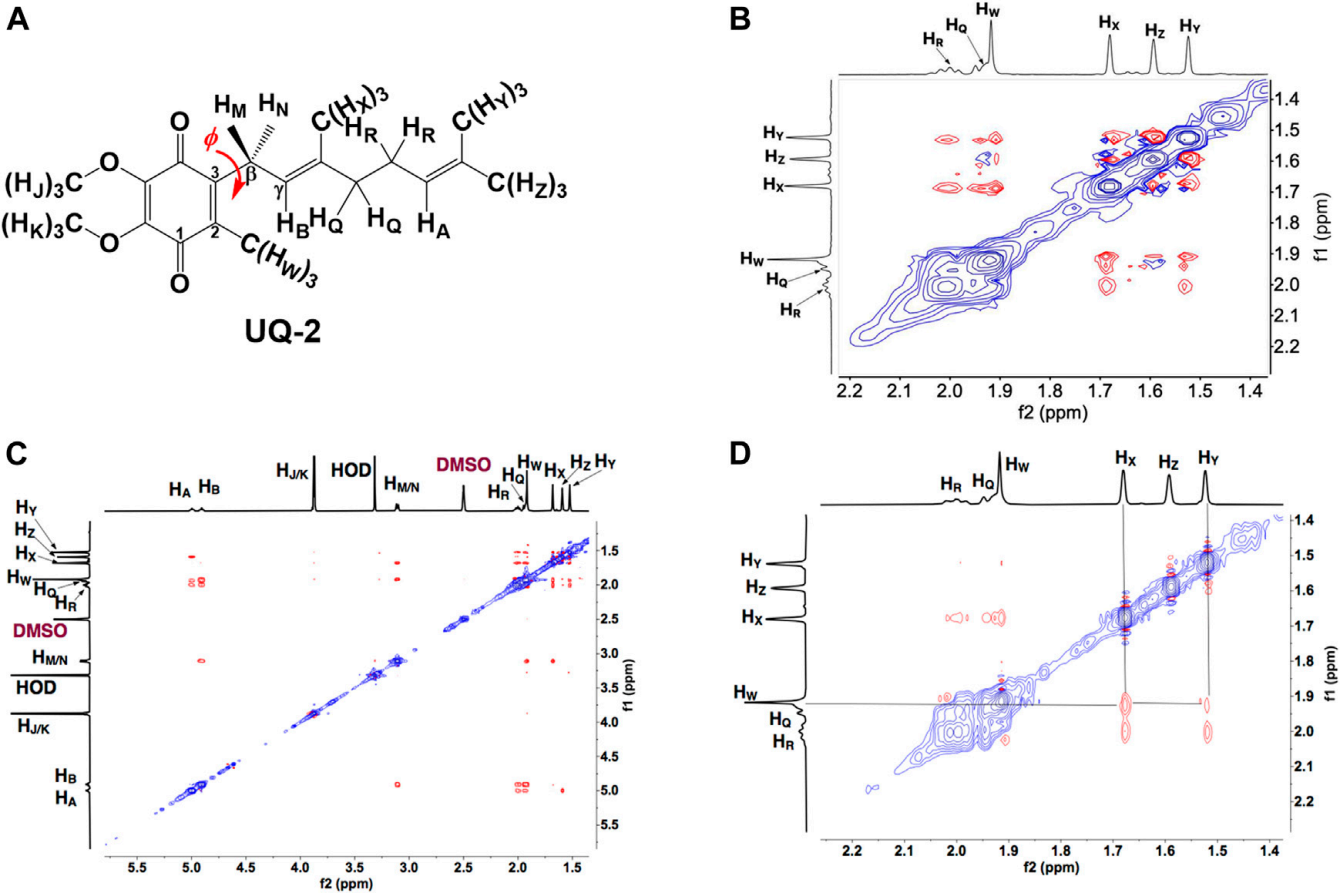
**(A)** Proton labeling scheme for UQ-2. **(B)** Partial ^1^H-^1^H 2D NOESY spectrum of UQ-2 in DMSO. **(C)** A larger ^1^H-^1^H 2D ROESY spectrum of UQ-2 in DMSO. Full ROESY spectrum can also be found in the Supplementary Material, Supplementary Figure S13. **(D)** Partial ^1^H-^1^H 2D ROESY spectrum of UQ-2 in DMSO.

**FIGURE 6:**
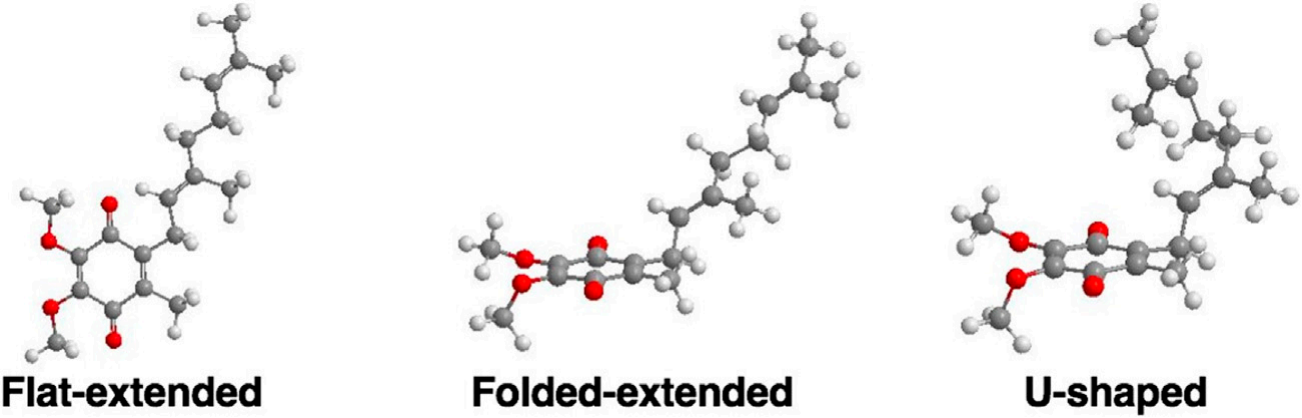
Examples of three possible classes of UQ-2 conformations: Flat-extended, Folded-extended, and U-shaped (Koehn et al., 2018b; Koehn et al., 2019).

To determine the conformation of UQ-2 in organic solvents, we utilized two complementary 2D NMR methods, ^1^H-^1^H 2D NOESY and ^1^H-^1^H 2D ROESY (Jones et al., 2011). We chose to highlight the NOESY and ROESY spectra of UQ-2 in d_6_-DMSO for direct comparison to our previous work with MK-2 (Koehn et al., 2018b). The corresponding spectra for d_3_-acetonitrile, d_6_-benzene, and d_5_-pyridine can be found in Supplementary Figures S15–S23 in the Supplementary Material. Looking at the structure of UQ-2, a folded conformation, which is defined by a ∼90° dihedral angle about the C2C3CβCγ bond, would be indicated by cross peaks between the headgroup and particular protons on the isoprenyl side chain such as the methyl protons H_W_ and protons further down the side chain, such as H_A_, H_Y_, and H_Z_. In the full NOESY spectrum in d_6_-DMSO, there is a cross peak observed between H_W_ and vinyl protons, H_A_ and H_B_ (Figure 4 and proton labeling scheme is shown in Figure 5A). The proton H_B_ is close enough to H_W_ have NOE interactions; however, H_A_ would be too far away to have NOE interactions unless the molecule is in a folded conformation. In addition, there are cross peaks that confirm the 1D ^1^H NMR spectra assignments along the isoprenyl tail, such as H_W_ and allylic protons H_M_ and H_N_ and between H_A_/H_B_ and methyl protons H_X_, H_Y_, and H_Z_. The folded conformation is also suggested by the cross peaks observed between methyl protons H_W_ and methyl protons, H_X_, H_Y_ and H_Z_ (Figure 5B). These cross peaks are also observed in the ROESY spectrum (Figures 5C,D. Enlarged full ROESY spectrum is also shown in the Supplementary Materials: Supplementary Figure S13). These cross peaks are indicative of a U-shaped conformation (example shown in Figure 6), placing the terminal methyl groups over the quinone headgroup for UQ-2 in d_6_-DMSO.

In the case of the 2D NOESY and ROESY spectra of UQ-2 in d_3_-acetonitrile (Supplementary Figures S16, S17), there are cross peaks observed between H_W_ and vinyl protons, H_A_ and H_B_, and methyl protons, H_X_, H_Y_, and H_Z_, similar to d_6_-DMSO. Together these suggest a folded, U-shaped conformation, but the results are somewhat inconclusive. The reference peak for d_3_-acetonitrile, 1.93 ppm, is close to H_W_ (1.94 ppm) and almost overlapping. Therefore, it is hard to conclude if the cross peaks observed are a result of intramolecular NOE interactions or interactions with the solvent. For 2D NOESY and ROESY spectra of UQ-2 in d_6_-benzene (Supplementary Figures S19, S20), there are cross peaks observed between H_W_ and H_X_, and H_M_/H_N_. In both the flat-extended and folded-extended conformations (example shown in Figure 6), H_X_, and H_M_/H_N_ are all within the 5 Å NOE range of H_W_. To distinguish between the two, we looked for cross peaks between H_W_ and the terminal methyl protons, H_Y_ and H_Z_. In the folded-extended conformation, the average distance between H_W_ and H_Y_ is approximately 5.1 Å. In the ROESY spectrum, there are cross peaks present between H_W_ and H_Y_ and H_Z_ (Supplementary Figure S20) and they are present in the corresponding NOESY (Supplementary Figure S19). Therefore, the presence of a cross peak between H_W_ and H_Y_ in the NOESY (Supplementary Figure S19) suggests H_Y_ is inside the NOE range and therefore indicates a folded, open U-shaped conformation in d_6_-benzene. Similar observations were found for 2D NOESY and ROESY spectra for UQ-2 in d_5_-pyridine (Supplementary Figures S22, S23). There are cross peaks observed between H_W_ and H_A_/H_B_, H_X_, and H_M_/H_N_, but there is a cross peak observed between H_W_ and H_Y_. Similar to benzene, this together suggests UQ-2 adopts a folded, open U-shaped conformation in d_5_-pyridine.

### 2.4 Illustrating UQ-2 Conformations Determined by NMR Using Molecular Mechanics

**FIGURE 7:**
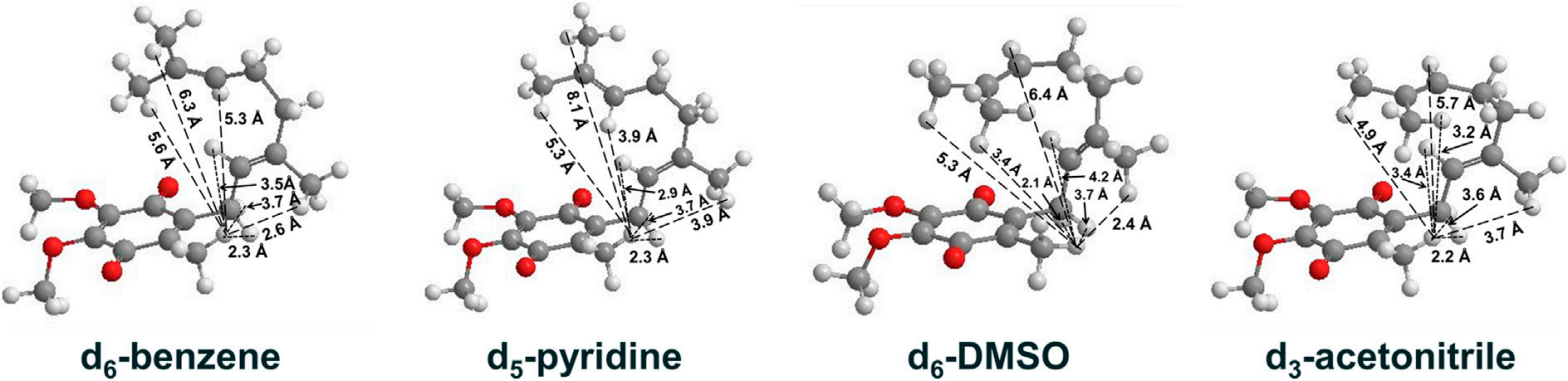
UQ-2 conformations generated using 2D NMR data distance constraints and MMFF94 calculations to illustrate the conformations elucidated in d_6_-benzene, d_5_-pyridine, d_6_-DMSO, and d_3_-acetonitrile. Intramolecular distances (Å) between H_W_ and select protons on the side chain are shown (dashed lines).

UQ-2 has a short repeating isoprenyl chain (C_10_) but enough carbons with numerous degrees of rotational freedom; therefore, even the truncated version of UQ-10, UQ-2, can assume many different specific conformations and still be considered folded by our definition. We created 3D conformations (Figure 7) of UQ-2 for visualization using Molecular Mechanics where intramolecular distances between specific protons obtained from 2D NOESY/ROESY NMR spectra (Supplementary Table S1) were used as geometric constraints. While the exact position of the isoprenyl side chain varies slightly from solvent to solvent, UQ-2 adopts a folded conformation in all four solvents examined in the 2D NMR studies, where the dihedral angle about the C2C3CβCγ bond (Figure 1C) is ∼90°.

To understand the differences between the conformations of UQ-2 and MK-2, we first had to determine the distances between the protons on the isoprenyl side chain and the headgroup methyl proton H_W_. We used the volume integrals from the 2D NOESY spectra and correlated them to intramolecular distances. The volume integral of the H_W_ and H_M_/H_N_ cross peak, which has a known intramolecular distance of ∼3.5 Å, was set to 1, and each volume integral was standardized against it. From here, the values obtained were used to determine if the cross peaks were strong (>1.5), medium (0.6–1.5), or weak (<0.6). These intensities were translated to distance in Ångstroms accordingly: 1) strong (<3 Å), 2) medium (3–4 Å), and 3) weak (>4 Å). The intramolecular distances were then used to construct the conformations, and MMFF94 minimization calculations were performed to correct bond lengths. The distances in Supplementary Table S1 reflect the intramolecular distances post minimization.

DMSO and pyridine were chosen due to the distinctly different 1D ^1^H NMR spectra to better illustrate the differences in conformation (Koehn et al., 2018b). Since NOESY and ROESY spectra are products of an average conformation, the intramolecular distances generated are representative of the most abundant conformation. As described previously, the interactions between headgroup methyl protons H_W_ and protons along the isoprenyl side chain, such as vinyl protons H_A_ and methyl protons H_X_, H_Y_, and H_Z_, are critical to evaluate UQ-2 conformation in organic solvents. The distances between H_W_ and H_A_, H_X_, H_Y_, and H_Z_ imply there is a folded conformation about the C2C3βCγ bond, and the position of the terminal methyl protons H_Y_ and H_Z_ relative to H_W_ suggest differences in the position of the end of the isoprenyl side chain: U-shaped or folded-extended conformation.

For UQ-2 in d_5_-pyridine, the intramolecular distance between H_W_ and H_X_ is shown to be 3.9 Å. The distance between H_W_ and vinyl proton, H_A_, 3.9 Å, supports a folded conformation. The intramolecular distances between H_W_ and H_Y_ and H_Z_ were found to be 8.1 Å and 5.3 A, respectively. Although these values are outside the range of NOE influence, cross peaks were observed in the 2D NOESY and ROESY spectra (Figures 4, 5C, respectively). Therefore, we cannot preclude the possibility that UQ-2 adopts both a folded-extended or a more open U-shaped conformation in d_5_-pyridine. These results are in line with MK-2 in d_5_-pyridine, which was previously found to adopt a folded-extended conformation (Koehn et al., 2018b).

**FIGURE 8:**
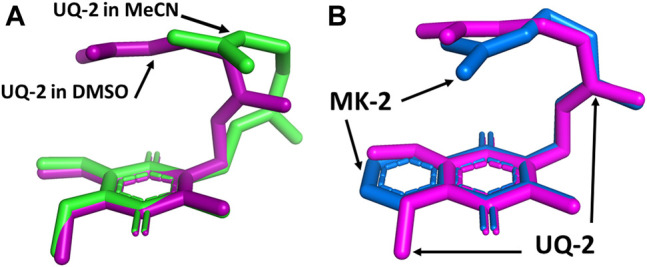
Superimposed 3D conformations of **(A)** UQ-2 based on the 2D NMR data in d_3_-acetonitrile (green) and d_6_-DMSO (purple) and **(B)** UQ-2 (pink) and MK-2 (blue) based on the 2D NMR data in d_6_-DMSO.

We superimposed the conformations of UQ-2 in d_3_-acetonitrile and d_6_-DMSO to better visualize the minute differences observed between conformations (Figure 8). The superimposed conformations of UQ-2 in d_6_-benzene and d_5_-pyridine are found in the Supplementary Material (Supplementary Figure S25). In Figure 8A, UQ-2 in d_3_-acetonitrile and d_6_-DMSO are shown in green and purple, respectively. With the headgroups aligned, there is a slight variation in the dihedral angle along the C2C3CβCγ bond, and the trend continues along the sidechain through the second isoprene unit. This accounts for the differences observed in intramolecular distances toward the end of the isoprenyl side chain. The terminal methyl groups of UQ-2 in d_6_-DMSO appear directly above the headgroup leading to a U-shaped conformation. The same methyl groups in d_3_-acetonitrile appear to be above but not centered over the headgroup, which is consistent with the possibility of U-shaped or folded-extended conformation. In Figure 8B, the conformation of UQ-2 and MK-2 in d_6_-DMSO are superimposed to visualize how the headgroup structure affects the conformation. The C2C3CβCγ bond in both UQ-2 and MK-2 are nearly identical to one another, but the conformation along the side chain starts to deviate past the first alkene. Taking into consideration the many degrees of freedom about the isoprenyl side chain, it is not unexpected to see deviations in conformation along the side chain.

The folded conformations observed for UQ-2 are likely a result of non-covalent interactions. Firstly, UQ-2 is folding upon itself to minimize interactions with the solvent due to the hydrophobic effect. This is especially apparent in the U-shaped conformations of UQ-2 in the hydrophilic solvents, DMSO and acetonitrile, whereas the hydrophobic solvents, pyridine and benzene, afford an open U-shaped or folded-extended conformation. Additionally, the folded conformation is likely reinforced by π-π interactions between the π bonds in the quinone headgroup and the isoprenyl side chain. This idea is supported by the work done on farnesol, where farnesol does not adopt a folded conformation, regardless of the increased number of degrees of freedom along the molecule (Zahn et al., 2000). Therefore, the presence of the quinone headgroup plays a significant role in the ability of the lipoquinone to adopt a folded conformation, but we only saw modest differences between the folded UQ-2 and MK-2 conformations. In addition to the presence of the lipoquinone headgroup, a study suggests the methyl proton, H_W_, on the quinone also plays an important role in influencing a folded conformation (Eddine et al., 2020). The quinone methyl group serves as a rotational barrier by preventing the side chain from freely rotating between folded and flat conformations.

### 2.5 1D ^1^H NMR Spectroscopic Studies of UQ-2 in RMs

**FIGURE 9:**
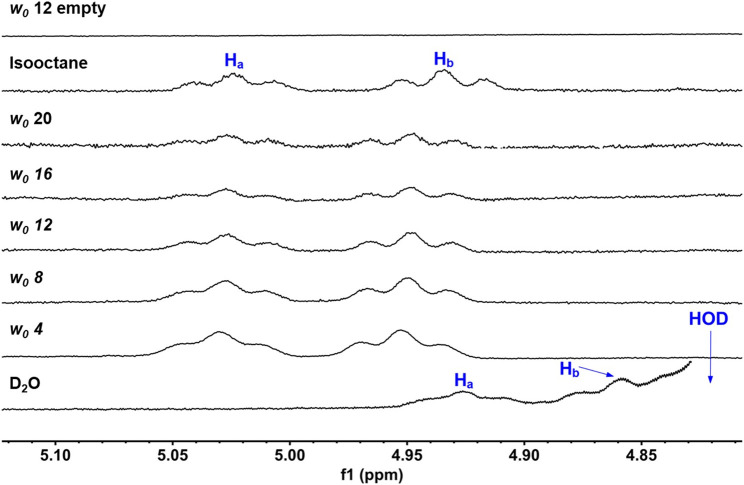
1D ^1^H NMR (400 MHz) spectra of UQ-2’s alkene protons, H_A_ and H_B_, in D_2_O, isooctane, and different sized RMs. An empty RM lacking UQ-2 is also shown for comparison. Peak labelling corresponds to the proton labelling scheme key in Figure 5A.

Our ultimate objective was to determine how UQ-2 behaved with respect to membrane-like interfaces. We used a system comprised of nanosized water droplets encased in AOT surfactant to create reverse micelles (RMs) inside an isooctane (hydrophobic) solvent. This simple model system will provide useful information regarding location, orientation, and conformation of UQ-2 at an interface using NMR spectroscopy with minimal overlap between surfactant proton peaks and key UQ-2 proton peaks. 1D ^1^H NMR spectra of varying RM sizes were collected in D_2_O (*w*_*0*_: 4, 8, 12, 16, 20, where *w*_*0*_ = [D_2_O]/[AOT]), shown in Figure 9. The placement of the UQ-2 molecules inside the membrane system was determined via the changes in chemical shift that UQ-2 protons undergo as the RM size changes. Typically, aromatic protons often offer ideal peaks to compare and analyze shifts, as they lie far from any AOT peaks. However, as there are no aromatic protons to observe in UQ-2, we instead observed the vinyl protons peaks (H_A_/H_B_), which are both triplet peaks that are easily discernable from the AOT peaks. The peak locations for H_A_ and H_B_ respectively are 5.02 and 4.93 ppm for isooctane, 5.03 and 4.95 ppm for *w*_*0*_ 20, 5.03 and 4.95 ppm for *w*_*0*_ 16, 5.03 and 4.95 ppm for *w*_*0*_ 12, 5.03 and 4.95 ppm for *w*_*0*_ 8, 5.03 and 4.95 ppm for *w*_*0*_ 4, 4.93 and 4.86 ppm for D_2_O. The chemical shifts of these peaks do not change significantly enough as the RM changes in size to reliably indicate where in the RM interface the UQ-2 resides. However, the large shift between the D_2_O sample and the RM samples (−0.10 ppm for H_A_ and −0.09 ppm for H_B_ from *w*_*0*_ 4 to D_2_O) compared to the much smaller shift between the isooctane sample and the RM samples (0.01 ppm for H_a_ and 0.02 ppm for H_b_ from isooctane to *w*_*0*_ 20) would indicate that the UQ-2 does not reside within the bulk water or in the isooctane of the RM system, and thus UQ-2 must reside somewhere in the RM interface. However, 2D NMR studies will enable the exact location, orientation, and conformation of UQ-2 in RMs to be identified.

Dynamic Light Scattering (DLS) was used to determine the hydrodynamic radius of RMs containing UQ-2 and verify that RMs formed in the samples used and that the properties and stability of the samples were consistent with previous studies (see Section V of the Supplementary Material). DLS samples were prepared following the sample preparation method used for NMR spectroscopic studies except that DDI H_2_O was used instead of D_2_O. The results showed that RMs formed and that the sizes of the RMs made were in excellent agreement with that of the literature (Maitra, 1984). These results indicate that the introduction of UQ-2 into the RM system does not significantly affect the size or stability of the RMs.

### 2.6 ^1^H-^1^H 2D NOESY NMR Spectroscopic Studies of UQ-2 in a RM Model Membrane System

**FIGURE 10:**
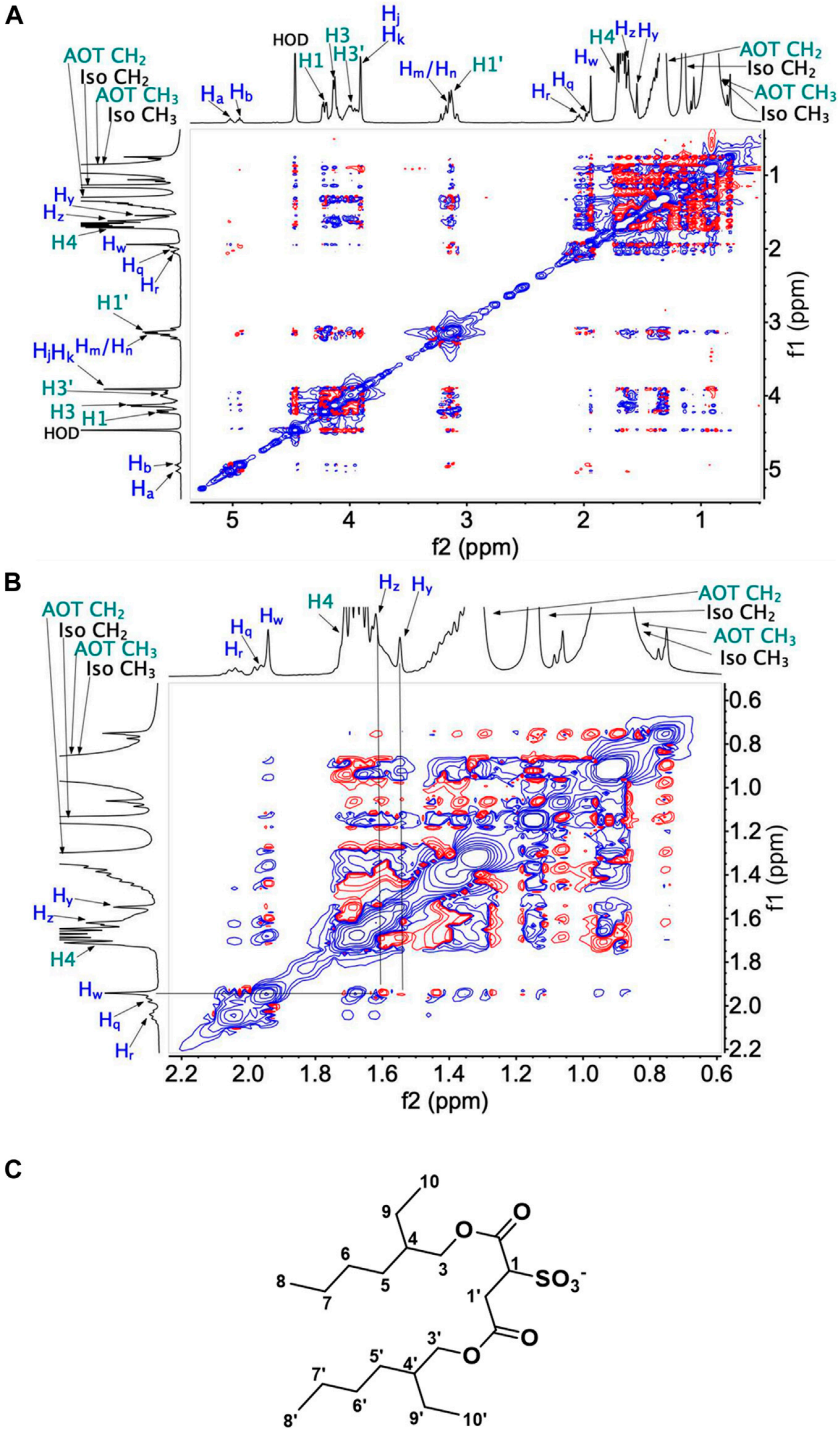
^1^H-^1^H 2D NOESY NMR spectra of UQ-2 in *w*_*0*_ 12 AOT-RM: **(A)** full spectrum, **(B)** partial spectrum. **(C)** Labeled structure of AOT. Blue text labels correspond to UQ-2 protons and teal text labels to AOT protons.

**FIGURE 11:**
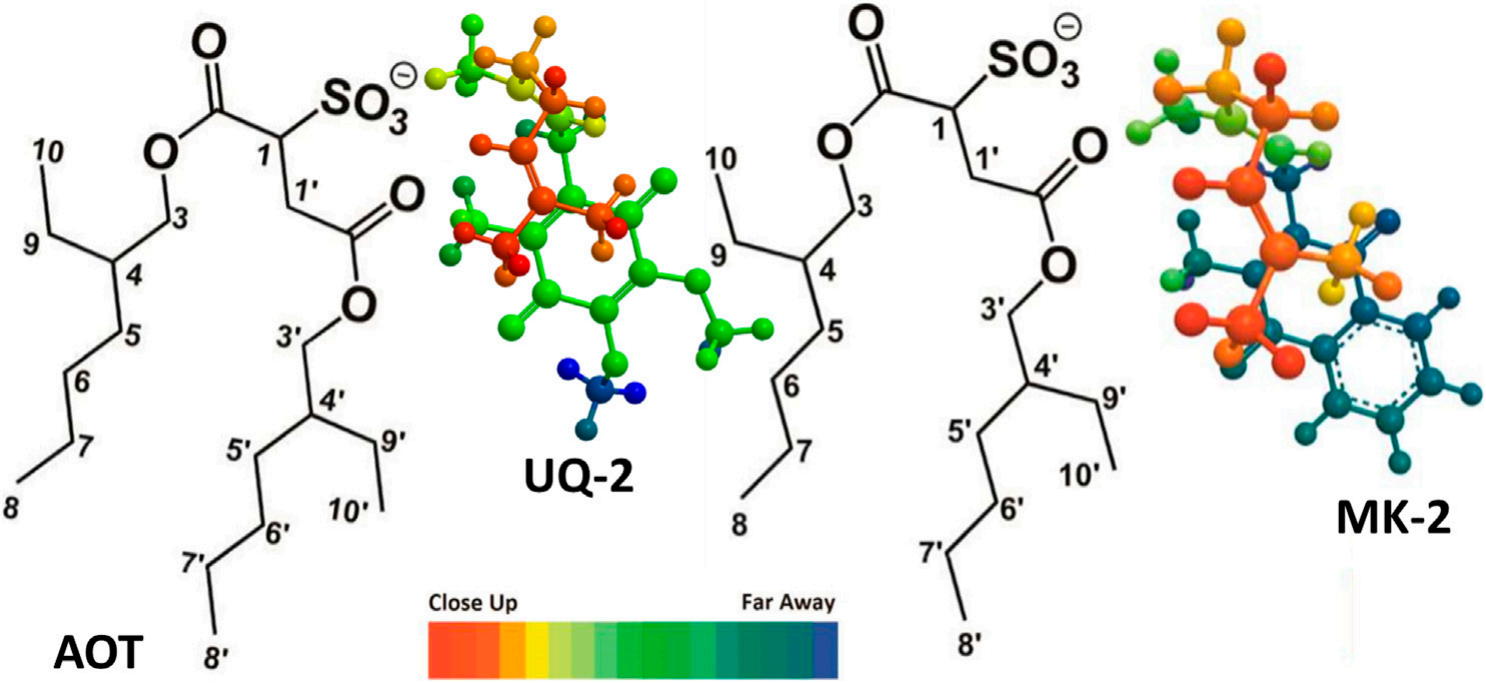
Illustration of UQ-2 and MK-2’s proposed folded, U-shaped conformations and placement within the RM interface. The arrangements are consistent with ^1^H-^1^H 2D NOESY and ROESY NMR spectral data obtained in a *w*_*0*_ 12 RM. Color depth gradient shows dark red as closer in distance and the dark blue as farther in distance from the reader. AOT proton labeling scheme key is shown.

To determine the exact location, orientation, and conformation of UQ-2 within RMs, we obtained an ^1^H-^1^H 2D NOESY NMR of UQ-2 in a *w*_*0*_ 12 RM (Figure 10). Figure 10A shows a full NOESY spectrum. To elucidate the location and orientation of UQ-2 within the RM, we looked for the interactions of UQ-2 with AOT (Figure 10C). Methoxy protons H_J_ and H_K_ and benzoquinone methyl protons H_W_ are shown to have cross peaks with AOT between H1′, H4, AOT-CH_2_, and AOT-CH_3_. Additionally, H_W_ shows cross peaks with H1, H3’, and H3 of AOT. Vinyl protons H_A_/H_B_ were found to have cross peaks with H1, H3, and H3’. Allylic protons H_M_/H_N_ were found to have interactions with H1, H3, H3′ and AOT-CH_2_, and H_Q_/H_R_ were found to have interactions with H1′. Although UQ-2 is less hydrophobic than MK-2, it is still shown to penetrate the AOT-water interface. The 2D NOESY cross peaks illustrate that UQ-2 is positioned near the interface of AOT with the methoxy groups of the headgroup oriented towards the alkyl chains of AOT (Figure 11). This orientation is similar to that of MK-2 (Koehn et al., 2018b), however the interactions between H_W_ and H_J_/H_K_ with H1-H4 indicate the molecule is positioned closer to the interface than MK-2, which is in agreement with our previous work showing the UQ headgroup is closer to the water pool than MK-2 in phospholipid bilayers (Koehn et al., 2018b; Van Cleave et al., 2020; Van Cleave et al., 2021). The cross peaks observed between H_W_ and H_Y_/H_Z_ and H_A_/H_B_ indicate UQ-2 is in a folded conformation, specifically a U-shaped conformation. This conformation would not be possible unless the isoprenyl side chain was positioned over the headgroup (Figure 10B). Using methods described above in Section 2.4, the interproton distances of UQ-2 in a *w*_*0*_ 12 AOT-RM system were determined between H_W_ and protons along the sidechain (Supplementary Table S2). The intramolecular distance between H_W_ and H_X_ is shown to be 2.6 Å. Additionally, the distance between H_W_ and H_Y_ and H_Z_ were found to be 4.6 Å and 3.7 Å, respectively. Together this supports a U-shaped conformation for UQ-2 in AOT-RMs. As a confirmatory measure, 1D NOE experiments were performed to confirm the interactions of H_A_/H_B_ with H_W_, H_Z_ and H1′ within the *w*_*0*_ 12 AOT-RM system (Supplementary Figure S24). Additionally, the 2D NOESY NMR experiment in AOT-RMs was repeated multiple times, and the same conclusions were made regarding location, orientation, and conformation of UQ-2 in the interface of the AOT-RM system.

### 2.7 Interaction of UQ-2 With Langmuir Phospholipid Monolayers

#### 2.7.1 Compression Isotherms of Pure and Mixed Monolayers

**FIGURE 12:**
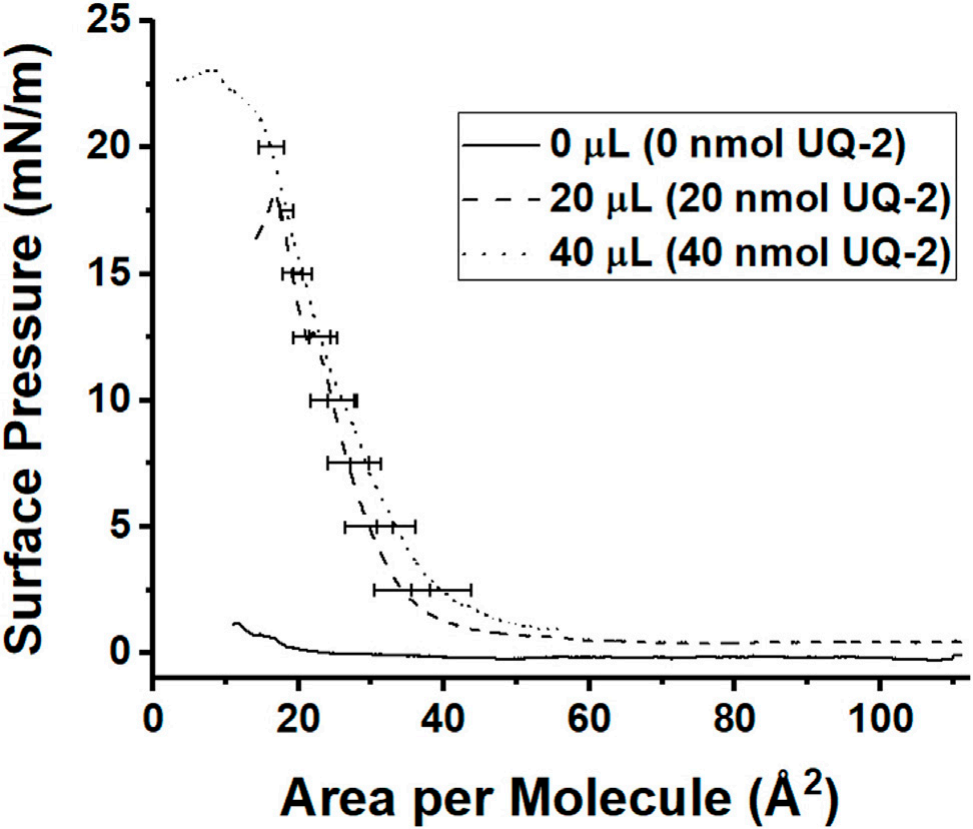
Compression isotherms of different amounts of 1 mM UQ-2. The solid black curve represents pure phosphate buffer with no UQ-2 present, the dashed curve represents an injection of 20 µl of UQ-2, and the dotted curve represents an injection of 40 µl of UQ-2. Curves are the average of at least three measurements. Standard deviations of the area were calculated for every 5 mN/m of surface pressure.

Finally, we wanted to understand how UQ-2 interacted with the interface of a phospholipid-based membrane monolayer and compare these results to the RM system. UQ-2 was found to have a collapse pressure of 21 mN/m in this study (Figure 12). Our value was obtained by taking the second derivative of area per molecule with respect to surface pressure, with the lowest point representing the collapse pressure. Reported literature values varied widely, with some as high as 35 mN/m and others reporting that UQ-2 dissolved into the subphase (Quinn and Esfahani, 1980; Bernard et al., 2000; Hoyo et al., 2017). These discrepancies may be due to the slightly soluble nature of UQ-2, differences in the composition of the subphase, and even stirring of the subphase.

Pure films of DPPC and DPPE were in line with the literature (Patterson et al., 2016; Hoyo et al., 2017), with DPPC exhibiting its signature gas-liquid transition between 0 and 5 mN/m and its liquid-liquid condensed transition from 5 to 10 mN/m of surface pressure. The gas-liquid transition disappears upon the addition of UQ-2. The normalized 75:25 DPPC:UQ-2 curve does not overlap with the control curve. However, the variability in our measurements makes it impossible to draw a solid conclusion on whether UQ-2 is compressed out of the DPPC monolayer. Regardless, compression modulus analysis showed that increasing amounts of UQ-2 caused the DPPC monolayer to become more elastic (Supplementary Figure S26).

**FIGURE 13:**
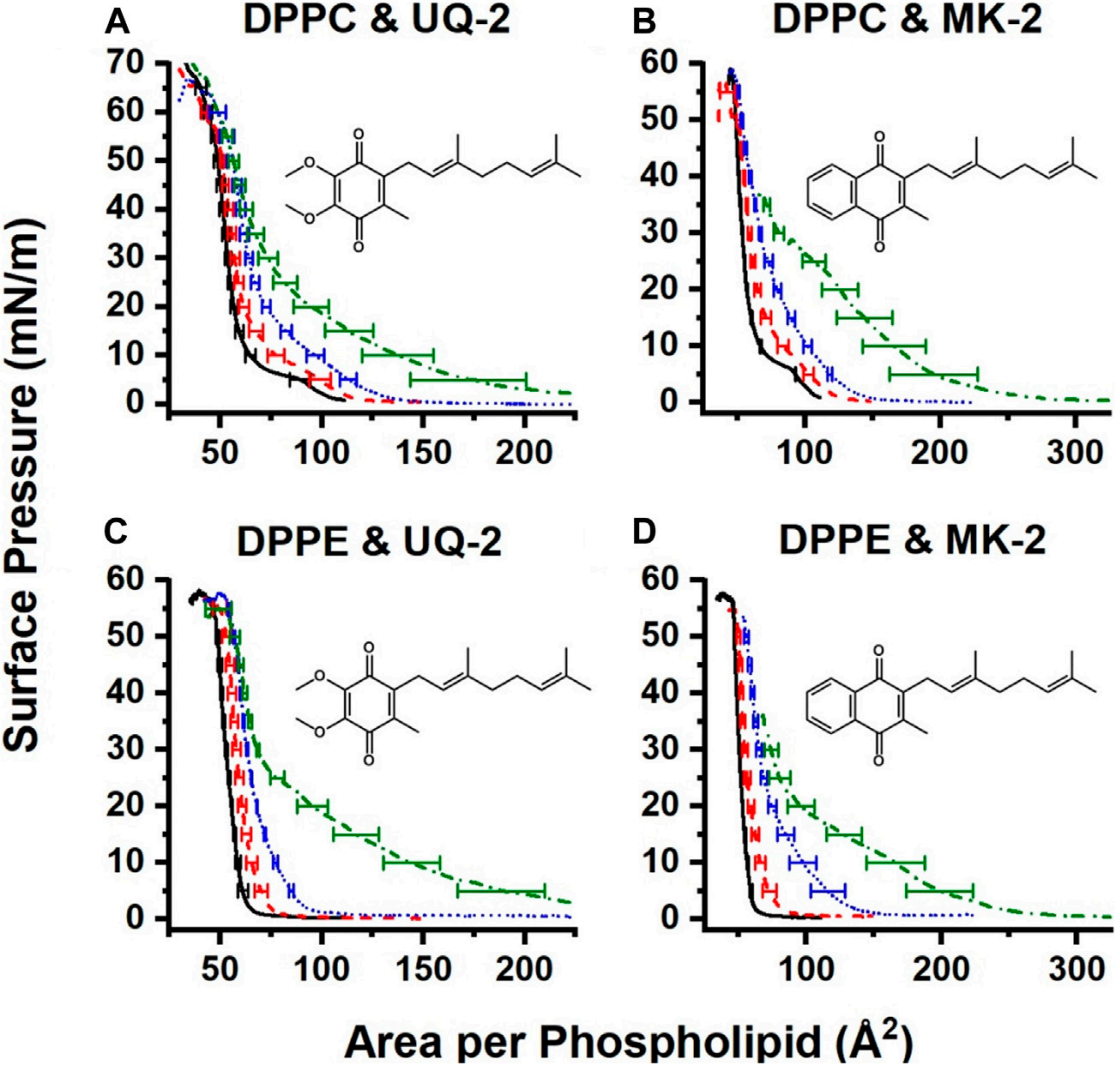
Normalized compression isotherms of mixed monolayers of UQ-2 **(A and C)** or MK-2 **(B and D)** and either DPPC **(A and B)** or DPPE **(C and D)**. Solid black curves represent pure phospholipid monolayers, red dashed curves represent 75:25 lipid:lipoquinone films, blue dotted curves represent 50:50 lipid:lipoquinone films, and green dash-dot curves represent 25:75 lipid:lipoquinone films. Curves are the average of at least three measurements. Error bars are the standard deviation of the area at every 5 mN/m of surface pressure. MK-2 data were reproduced with permission from Koehn et al. 2018b).

Compression isotherms with DPPE did not exhibit a significant difference in collapse pressure, as seen in Figure 13. The 25:75 and 50:50 DPPE:UQ-2 monolayers exhibited a liquid condensed phase, which suggests a reorganization of the monolayer. All mixed DPPE monolayers demonstrated at least a 9% increase in area per molecule at physiological surface pressure (Jones and Chapman, 1995) (30–35 mN/m) without the normalized curves overlapping with the control (see Supplementary Tables S3, S4). This indicates that UQ-2 is in the interface and spreading the DPPE molecules apart. UQ-2 was not compressed out of the monolayer at physiological surface pressure for DPPE.

#### 2.7.2 Comparison of UQ-2 and MK-2 in Langmuir Monolayers

Interestingly, UQ-2 remains in the DPPC interface at physiological surface pressure, whereas MK-2 was likely to be compressed out. There is also a more distinct hump in the 25:75 DPPC:MK-2 curve at 25 mN/m than that of the 25:75 DPPC:UQ-2. In combination with the fact that mixed DPPC:UQ-2 curves do not overlap the control; we might conclude that UQ-2 is more likely to reside in the interface than MK-2. This is logical, as MK is more hydrophobic than UQ.

Both UQ-2 and MK-2 induce a liquid-condensed phase in DPPE, but it appears with a smaller mole fraction of MK-2 than UQ-2. As with DPPC, UQ-2 is always present in the interface as the mixed monolayers are at least 9% more expanded at physiological surface pressure than the DPPE control. Again, the hydrophobicity of the naphthoquinone headgroup of MK-2 is greater than that of the benzoquinone headgroup of UQ-2 which likely explains these differences. In summary, both UQ-2 and MK-2 associated with the Langmuir monolayer interface, but UQ-2 resided closer to the interfacial water than MK-2 due to MKs more hydrophobic nature, consistent with the RM model membrane studies.

## 3 Conclusion

UQs and MKs are critical components of the ETS. UQs are found in bacteria, fungi, plants, and mammals, and MKs are mainly found in gram positive bacteria. While the interactions of these electron transport lipids with the membrane-bound protein complexes along the ETS is well-known, surprisingly their exact location and conformation within the membrane is still widely debated. In this study, we determined the location, orientation, and conformation of UQ-2, a truncated, representative analog for native UQ-10, using 1D and 2D NMR spectroscopic methods in organic solution and biological membrane-like environments. We then compared the UQ-2 results to the previously studied MK-2 to understand fundamentally how the class of lipoquinone molecules behave within membranes.

The 1D and 2D NMR studies showed that different solution environments slightly change the observed folded conformation of UQ-2. In all four solvents examined in this study (DMSO, acetonitrile, pyridine, and benzene), UQ-2 was found to adopt a folded, U-shaped conformation with a ∼90° dihedral angle about the C2C3CβCγ. On the other hand, UQ-2 adopts a more open U-shaped conformation in the hydrophobic solvents, benzene and pyridine, which documents the fact that the environment will impact the conformation of the UQ-2 side chain. Once we established that UQ-2 folded in solution, we wanted to determine if a folded conformation was also adopted in a membrane-like interface. Using 2D NMR spectroscopy, we determined that UQ-2 interacts similarly to MK-2 with the RM model membrane system. Both UQ-2 and MK-2 adopt a folded, U-shaped conformation but reside at slightly different places in the membrane-like interface. Not surprisingly, and consistent with other studies (Van Cleave et al., 2020; Van Cleave et al., 2021), UQ-2 resides closer to the AOT-water interface than the more hydrophobic MK-2. Both lipoquinone molecules were oriented in a manner that allowed the side chain to fold back over the quinone moiety and be accommodated in the surfactant tails. It appears that regardless of lipoquinone headgroup structure, lipoquinones adopt folded conformations at membrane-like interfaces. Langmuir monolayer studies examining the interaction of UQ-2 with DPPC and DPPE phospholipids supported the results of the RM studies. Both UQ-2 and MK-2 were found to associate with the monolayer water-lipid interface, but MK-2 was more easily compressed out of the interface, which indicates UQ-2 resides closer to the interface than MK-2.

In summary, lipoquinones UQ-2 and MK-2 adopted folded conformations in solution and within membrane-like interfaces. The more polar UQ-2 was found to reside slightly closer to the water-surfactant interface, which was supported by both the RM and Langmuir monolayer studies. It appears that the presence of a lipoquinone headgroup is important for anchoring the lipoquinone in the membrane interface and for allowing the isoprenyl side chain to adopt some variation of a folded conformation that can be accommodated within the lipid tails due to the orientation of the lipoquinone within the interface. Varying the structure of the lipoquinone (UQ vs. MK) only modestly changed the location while residing in the RM interface. However, since lipoquinones are redox-active and the polarity of the headgroup changes upon reduction to the quinol form, structural differences in the headgroup likely facilitate locomotion of headgroup within the interface between membrane-bound enzymes in the ETS (Van Cleave et al., 2020). Taken together, the results of this study and others support a model where the headgroups of the longer, native lipoquinones, such as UQ-10 and MK-9, reside close to the water-lipid interface with the side chains folded but penetrating through the acyl tails into the midplane of the membrane bilayer. The location, orientation, and conformation of lipoquinones are critical for their function in generating cellular energy within membrane ETS and the studies described herein shed light on the behavior of lipoquinones within membrane-like environments.

## 4 Experimental

### 4.1 General Materials

The following chemicals were used without further purification for the synthetic work: Ultra-high purity argon (99.9%, Airgas), 3,4,5-trimethoxytoluene (97%, Aldrich), α,α-dichloromethyl methyl ether (98%, Aldrich), Dichloromethane (DCM, Stabilized, 99.9%, Fisher Scientific), TiCl_4_ (99.9%, Aldrich), *n-*pentane (98%, Merck), Ethyl Acetate (99.9%, Fisher), Diethyl ether (≥99.0%, Merck), NaHCO_3_ (99.7%, Merck), NaCl (Fisher), Na_2_SO_4_ (Fisher), Methanol (Aldrich), 30% aq. H_2_O_2_ solution (Sigma-Aldrich), H_2_SO_4_ (Fisher), SiO_2_ (SiliCycle^®^ SilicaFlash^®^ F60, 43–60 μm 60 Å), 60% NaH dispersion in mineral oil (Aldrich), THF (Fisher), Geranyl Bromide (95%, Aldrich), NH_4_Cl (99.7%, Fisher), BF_3_ diethyl etherate (≥46.5%, Aldrich), MgSO_4_ (98%, Merck), Acetonitrile (99.9%, Fisher) and FeCl_3_ hexahydrate (99.9%, Fisher). The following chemicals were used without further purification for the spectroscopic studies: D_2_O (99.9%, Cambridge Isotope Laboratories), d_1_-chloroform (99.8%, Cambridge Isotope Laboratories), d_3_-acetonitrile (99.8%, Aldrich), d_6_-DMSO (99.9%, Cambridge Isotope Laboratories), d_5_-pyridine (99.8%, Merck), d_6_-benzene (99.5%, Cambridge Isotope Laboratories), isooctane (99.8%, Aldrich), and AOT (≥99%, Aldrich). The following materials were used for the trough work: Sodium phosphate monobasic monohydrate (≥98%) and chloroform (≥99.8%) were purchased from Sigma Aldrich. Sodium phosphate dibasic anhydrous (≥99%) and methanol (≥99.9%) were purchased from Fisher Scientific. 1,2-dipalmitoyl-*sn*-glycero-3-phosphocholine (DPPC, >99%) and 1,2-dipalmitoyl-sn-glycero-3-phosphoethanolamine (DPPE, >99%) were purchased from Avanti Polar Lipids. Distilled deionized water (DDI H_2_O) was purified with a Barnsted E-pure system (∼18 MΩ-cm).

### 4.2 General Methods

All reactions were carried out under argon atmosphere unless otherwise noted. All reagents were used as purchased unless otherwise noted. Solvents were dried by passing through an alumina drying column (Solv-Tek Inc.) under argon pressure (DCM, THF, diethyl ether).

### 4.3 Syntheses

#### 4.3.1 Preparation of 2,3,4-Trimethoxy-6-Methylbenzaldehyde (2)

To a dry 500 ml round bottom Schlenk flask was added dry dichloromethane (DCM) (150 ml) followed by 3,4,5-trimethoxytoluene **1** (9.97 g, 54.7 mmol) and α,α-dichloromethyl methyl ether (12.58 g, 109.5 mmol, 2 eq.) and then cooled to 0°C. Then, TiCl_4_ (273.6 mmol, 137 ml, 2.0 M in dry DCM, 2.5 eq. to α,α-dichloromethyl methyl ether) was added dropwise over 30 min via a 250 ml addition funnel under argon at 0°C. After addition was complete, the red reaction mixture was stirred at ambient temperature for 20 h. Thin layer chromatography (TLC) (9:1 *n*-pentane/EtOAc) showed the reaction was complete. The reaction was then very slowly quenched with ice until the reaction color turned light blue-gray. The DCM was removed under reduced pressure at ambient temperature and the resulting off-yellow liquid was extracted with diethyl ether (3 × 100 ml). The combined organic extracts were washed with sat. NaHCO_3_ (200 ml), washed with brine (3 × 100 ml), dried over anhydrous Na_2_SO_4_, and then the solvent was removed under reduced pressure at ambient temperature. The product was dried under reduced pressure (∼125 Torr) for 1 h, which yielded an off-white crystalline solid (11.40 g, 54.2 mmol, 99.1%) that was pure. ^1^H NMR (400 MHz, CDCl_3_) δ: 10.40 (s, 1H), 6.50 (s, 1H), 3.98 (s, 3H), 3.92 (s, 3H), 3.86 (s, 3H), 2.56 (s, 3H). ^13^C NMR (101 MHz, CDCl_3_) δ: 191.18, 158.46, 157.78, 139.80, 138.26, 121.48, 110.53, 62.50, 61.12, 56.15, 21.91. HRMS (ESI, OTOF) m/z: [(M + H)^+^] Calcd for C_11_H_15_O_4_ 211.0965; Found 211.0965.

#### 4.3.2 Preparation of 2,3,4-Trimethoxy-6-Methylphenol (3)

To a 250 ml round bottom Schlenk flask was added 2,3,4-trimethoxy-6-methylbenzaldehyde **2** (11.37 g, 54.1 mmol), MeOH (110 ml), and 30% aq. H_2_O_2_ solution (7.97 g, 70.3 mmol, 1.3 eq.). Then, conc. H_2_SO_4_ (1.08 ml) was added dropwise resulting in a red-orange reaction mixture. After 2.5 h of stirring at ambient temperature under argon, the ^1^H NMR spectrum showed no aldehyde peak present. The reaction mixture was poured into sat. NaHCO_3_ (100 ml) and then extracted with DCM (3 × 100 ml). The combined organic extracts were washed sat. NaHCO_3_ (100 ml), washed with brine (100 ml), dried over anhydrous Na_2_SO_4_, and the solvent was evaporated under reduced pressure (∼125 Torr) at ambient temperature to yield 10.55 g crude red oil. The product was then purified by flash column chromatography (7:1 *n*-pentane/EtOAc, 600 ml SiO_2_, 70 mm column) to yield the desired product as an off-white crystalline solid (9.75 g, 49.2 mmol, 90.9%). ^1^H NMR (CDCl_3_) δ: 6.43 (s, 1H), 5.43 (s, 1H), 3.95 (s, 3H), 3.86 (s, 3H), 3.80 (s, 3H), 2.21 (s, 3H). ^13^C NMR (101 MHz, CDCl_3_) δ:146.17, 141.24, 140.14, 140.09, 118.08, 109.63, 61.34, 61.11, 56.75, 15.65. HRMS (ESI, OTOF) m/z: [(M + H)^+^] Calcd for C_10_H_15_O_4_ 199.0965; Found 199.0951.

#### 4.3.3 Preparation of Geranyl 2,3,4-Trimethoxy-6-Methylphenyl Ether (6)

NaH (2.95 g of 60% NaH dispersion in mineral oil washed with *n-*pentane that was first dried over activated neutral alumina, 73.8 mmol) was added to a dry 500 ml round bottom Schlenk flask containing a solution of 2,3,4-trimethoxy-6-methylphenol **3** (9.75 g, 49.2 mmol) dissolved in dry THF (150 ml). The mixture was stirred at ambient temperature for 35 min. The solvent was removed under reduced pressure at 25°C until an off-white powder was obtained. To a dry 500 ml round-bottom Schlenk flask was added the crude powder dissolved in anhydrous THF (75 ml) and then the mixture was cooled in an ice-H_2_O bath. Then, a solution of geranyl bromide (16.02 g, 73.8 mmol, 1.5 eq.) in dry THF (75 ml) was added dropwise over 15 min via an addition funnel under argon. The mixture was then warmed to ambient temperature and stirred for 21.5 h under argon. The resulting yellow reaction mixture was slowly quenched with 5% aq. NH_4_Cl solution (100 ml) and then extracted with diethyl ether (3 × 100 ml). The combined yellow organic extracts were washed with sat. NaHCO_3_ (100 ml), washed with brine (100 ml), dried over anhydrous Na_2_SO_4_, and then the solvent was removed under reduced pressure (∼125 Torr) at ambient temperature to yield 22.4 g crude yellow oil. The crude oil was purified by flash column chromatography (9:1 *n*-pentane/EtOAc, 1,200 ml SiO_2_, 70 mm column) to yield a light-yellow oil (9.75 g, 29.2 mmol, 59.4%). ^1^H NMR (CDCl_3_) δ: 6.44 (s, 1H), 5.55 (t, J = 7.2 Hz, 1H), 5.10 (t, J = 6.3 Hz, 1H), 4.45 (d, J = 7.1 Hz, 2H), 3.93 (s, 3H), 3.86 (s, 3H), 3.81 (s, 3H), 2.22 (s, 3H), 2.04–2.12 (m, 4H), 1.69 (s, 6H), 1.61 (s, 3H). ^13^C NMR (101 MHz, CDCl_3_) δ: 149.19, 147.42, 144.52, 141.48, 140.97, 131.81, 126.54, 124.11, 120.48, 108.45, 69.74, 61.37, 61.23, 56.28, 39.79, 26.53, 25.83, 17.83, 16.47, 16.43. HRMS (ESI, QTOF) m/z: [(M + H)^+^] Calcd for C_20_H_31_O_4_ 335.2217; Found 335.2220.

#### 4.3.4 Preparation of 5-Geranyl-2,3,4-Trimethoxy-6-Methyl-Phenol (7)

To a 500 ml round bottom Schlenk flask was added geranyl 2,3,4-trimethoxy-6-methylphenyl ether **6** (9.73 g, 29.1 mmol) dissolved in dry diethyl ether (120 ml). Then, the mixture was treated with fresh BF_3_ etherate (36.9 ml, 46.5% BF_3_ etherate solution, 291.0 mmol, 10 eq.) added dropwise over 5 min and then stirred under argon at ambient temperature for 30 min. After exactly 30 min, the brown colored reaction was quenched with sat. NaCl (100 ml) very slowly over 8 min and then the mixture was extracted with diethyl ether (3 × 100 ml). The combined light yellow organic extracts were washed with sat. NaHCO_3_ (50 ml, releases CO_2_), washed with brine (100 ml), dried over anhydrous MgSO_4_, vacuum filtered, and then the solvent was removed under reduced pressure (∼125 Torr) at ambient temperature to yield 9.41 g crude brown oil. The product was purified by flash column chromatography (9:1 *n*-pentane/EtOAc, 800 ml SiO_2_, 70 mm) to yield the desired product (4.67 g, 14.0 mmol, 48.1%) as a light-yellow oil. ^1^H NMR (CDCl_3_) δ: 5.60 (s, 1H), 5.05 (q, J = 6.5, 2H), 3.93 (s, 3H), 3.91 (s, 3H), 3.75 (s, 3H), 3.33 (d, J = 6.5, 2H), 2.13 (s, 3H), 2.07 (q, J = 7.2, 2H), 1.98 (m, 2H), 1.76 (s, 3H), 1.65 (s 3H), 1.57 (s, 3H). ^13^C NMR (101 MHz, CDCl_3_) δ: 144.49, 143.61, 143.44, 137.85, 135.12, 131.48, 129.82, 124.43, 123.18, 117.68, 61.44, 61.32, 60.92, 39.83, 26.78, 25, 82, 25.66, 17.82, 16.34, 11.48. HRMS (ESI, QTOF) m/z: [(M + Na)^+^] Calcd for C_20_H_31_O_4_Na 357.2036; Found 357.2004.

#### 4.3.5 Preparation of Ubiquinone-2 (8)

To a 50 ml round bottom flask was added 5-geranyl-2,3,4-trimethoxy-6-methylphenol 7 (0.137 g, 0.410 mmol), followed by DCM (2 ml) and ACN (2 ml) and then cooled to 0°C. An excess of FeCl_3_ (1.11 g, 4.10 mmol, 10 eq.) dissolved in ACN (4 ml) was then added. The open atmosphere mixture was stirred at 0°C for 35 min. Then, DDI H_2_O (30 ml) and sat. aq. NaHCO_3_ (30 ml) were added to the orange reaction mixture and then extracted with diethyl ether (3 × 100 ml). The combined organic extracts were washed with sat. NaHCO_3_ (50 ml), washed with brine (50 ml), dried over anhydrous Na_2_SO_4_, and then the solvent was removed under reduced pressure at ambient temperature to yield UQ-2 as a red oil and practically pure (0.125 g, 0.393 mmol, 95.9%). ^1^H NMR (CDCl_3_) δ: 5.03 (t, J = 6.8, 1H), 4.92 (t, J = 7.0, 1H), 3.99 (s, 3H), 3.98 (s, 3H), 3.18 (d, J = 7.0, 2H), 2.01 (m, 8H), 1.72 (s, 3H), 1.64 (s, 3H), 1.57 (s, 3H). ^13^C NMR (101 MHz, CDCl_3_) δ: 184.91, 184.05, 144.53, 144.38, 141.84, 139.01, 137.66, 131.68, 124.13, 199.08, 61.28, 39.82, 26.66, 25.81, 25.43, 17.82, 16.43, 12.07. HRMS (DART) m/z: [(M + H)^+^] Calcd for C_19_H_27_O_4_ 319.1904; Found 319.1935.

### 4.4 Mass Spectrometry

High resolution mass spectrometry (HRMS) experiments were carried out using one of the following instruments: 1) an Agilent 6220 TOF LC/MS (“OTOF”) interfaced to an Agilent 1200 HPLC, 2) a Maxis QTOF (“QTOF”) with electrospray (ESI) mode, and 3) a Maxis QTOF in positive DART mode (DART) using jeffamine as an internal calibration standard.

### 4.5 NMR Spectroscopic Studies

1D and 2D ^1^H studies were carried out both in organic solvents and a RM system. ^1^H and ^13^C spectra were recorded using either a Varian Model MR400 or Model Inova400 operating at 400 and 101 MHz, respectively. Chemical shift values (δ) are reported in ppm and referenced against the internal solvent peaks in ^1^H NMR (CDCl_3_, δ at 7.26 ppm; d_3_-acetonitrile δ at 1.94 ppm; d_6_-DMSO, δ at 2.50 ppm; d_6_-benzene, δ at 7.16 ppm; d_5_-pyridine, δ at 8.74 ppm; D_2_O, δ at 4.79 ppm) and in ^13^C NMR (d_6_-DMSO, δ at 39.52 ppm; d_6_-benzene, δ at 128.06 ppm). All NMR spectra were recorded at either 22°C or 26°C. When samples were prepared for RM NMR experiments, deuterium oxide was used instead of H_2_O, and the pH was adjusted to consider the presence of deuterium (pD = 0.4 + pH) (Samart et al., 2014).

For 1D and 2D NMR spectroscopic studies, a sample for analytical characterization of UQ-2 was prepared by using normal phase preparative thin layer chromatography (TLC) (10:1 *n*-pentane/EtOAc). First, ∼10 mg of UQ-2 (dissolved in minimal amount of DCM) was loaded onto a preparative TLC plate and then eluted (10:1 *n*-pentane/EtOAc, 45 min). The plate was briefly dried of eluent solvent and eluted a second time (10:1 *n*-pentane/EtOAc, 45 min). The orange band was illuminated under UV light and while illuminated, the band was divided into a top half and a bottom half. The bottom half was carefully removed with a razor blade, extracted with DCM, filtered through a disposable Pasteur pipette filled with glass wool (pre-rinsed with DCM) and concentrated under reduced pressure at ambient temperature to provide 6 mg of UQ-2 as a red oil for NMR spectroscopic studies.

#### 4.5.1 Solution 1D ^1^H NMR Spectroscopic Studies of UQ-2

Samples were prepared by dissolving 5.0 mg of UQ-2 in 0.5 ml of either d_1_-chloroform, d_6_-DMSO, d_5_-pyridine, d_3_-acetonitrile, and d_6_-benzene, respectively. The NMR instrument was locked onto the respective deuterium signal in the deuterated solvent used. NMR spectra were then collected using 32 scans for each sample. The data was processed using MestReNova NMR processing software version 10.0.1. The spectra were manually phased and then the baseline was corrected using a Bernstein Polynomial Fit (polynomial order 3). The obtained spectra were referenced to the internal solvent peak.

#### 4.5.2 Sample Preparation for ^1^H-^1^H 2D NOESY and ^1^H-^1^H 2D ROESY NMR Spectroscopic Studies of UQ-2

To prepare the samples in d_5_-pyridine, d_3_-acetonitrile, and d_6_-DMSO, 3.2 mg of UQ-2 was dissolved in 0.5 ml of solvent to yield a 20 mM solution of UQ-2. The NMR tubes containing the UQ-2 solution were purged with argon prior to data collection. To prepare a 100 mM solution of UQ-2 in CDCl_3_, 15.9 mg of UQ-2 was dissolved in 0.5 ml CDCl_3_. To prepare a 20 mM sample, 3.2 mg of UQ-2 was dissolved in 0.5 ml of each respective solvent (d_1_-chloroform, d_6_-DMSO, d_5_-pyridine, d_3_-acetonitrile, and d_6_-benzene).

#### 4.5.3 ^1^H-^1^H 2D NOESY and ^1^H-^1^H 2D ROESY NMR Spectroscopic Solution Experiments of UQ-2

^1^H-^1^H 2D NOESY NMR and ^1^H-^1^H 2D ROESY NMR spectroscopic experiments were conducted using a 400 MHz Varian MR400 NMR at 26°C. A standard NOESY pulse sequence was used consisting of 256 transients with 16 scans in the f1 domain using a 500 ms mixing time, 45° pulse angle, and a 1.5 s relaxation delay. A standard ROESYAD pulse sequence was used consisting of 256 transients with 16 scans in the f1 domain using a 400 ms mixing time, 45° pulse angle, and a 2.0 s relaxation delay. The NMR was locked onto either d_5_-pyridine, d_6_-benzene, d_3_-acetonitrile, or d_6_-DMSO. The resulting spectrum was processed using MestReNova NMR software version 10.0.1 (see Supplementary Material for details). The spectra were referenced to the internal solvent peak.

#### 4.5.4 Sample Preparation for RM NMR Spectroscopic Studies of UQ-2

A 0.50 M AOT stock solution was made by dissolving AOT (5.56 g, 12.5 mmol) in isooctane (25.0 ml). Empty RMs were made by mixing 0.50 M AOT stock solution with a D_2_O water pool, and then vortexed. UQ-2 RMs were made in a similar matter. The only difference being a 14.3 mM for UQ-2 stock solution was made by dissolving 45.4 mg of UQ-2 in 10.0 ml of 0.50 M AOT/isooctane solution. The RMs were then prepared using the UQ-2 stock solution. First, 2.0 ml samples were made using specific amounts of the 14.3 mM UQ-2 stock solution and then diluting the sample with the 500 mM AOT/isooctane solution. From the 2.0 ml solutions, 1.0 ml RM samples were prepared using the designated amounts of 2.0 ml sample and then adding the proper amount of D_2_O with pH 7.0 (see Section 4.2 for pH measurements) for UQ-2 to form the desired size RM. The samples were then vortexed until clear. The overall concentrations for 1.0 ml UQ-2 RM samples are as follows: *w*_*0*_ 4, 13.8 mM; *w*_*0*_ 8, 6.4 mM; *w*_*0*_ 12, 3.5 mM; *w*_*0*_ 16, 2.0 mM; and *w*_*0*_ 20, 1.4 mM.

#### 4.5.5 1D ^1^H NMR Spectroscopic Studies of AOT/Isooctane RMs Containing UQ-2

NMR spectra of various size RMs and in isooctane and D_2_O were obtained using a Varian Inova 400 MHz instrument at 22°C using routine parameters (pulse angle: 45°, relaxation delay of 1 s) using 64 scans. The NMR instrument was locked onto 10% D_2_O signal for the RM samples and D_2_O for the sample in D_2_O. The 1D ^1^H spectra of UQ-2 in isooctane were doped with 5% d_6_-benzene for the NMR instrument to lock onto and to achieve properly shimmed spectra. The spectral data was processed using MestReNova NMR processing software version 10.0.1. The spectra were manually phased and then the baseline was corrected using a multipoint baseline correction (cubic splines). The spectrum in D_2_O was referenced to the internal D_2_O peak and the spectra in isooctane and RM samples were referenced to the isooctane methyl peak (0.904 ppm) as previously reported (Samart et al., 2014).

#### 4.5.6 Sample Preparation for ^1^H-^1^H 2D NOESY and ROESY NMR Spectroscopic Studies of UQ-2 in AOT/Isooctane RMs

A 0.50 M AOT stock solution was made by dissolving AOT (5.56 g, 12.5 mmol) in isooctane (25.0 ml). A 1 ml stock solution of 112 mM UQ-2 in AOT/isooctane was made by dissolving 35.7 mg of UQ-2 in 1 ml isooctane/AOT stock solution. To make a *w*_*0*_ 12 RM, 893 μl of 112 mM UQ-2 AOT/isooctane stock solution and 107 μl of D_2_O at pH 7 were mixed together and then vortexed. This final mixture results in a *w*_*0*_ 12 RM microemulsion with an overall concentration of UQ-2 being ∼100 mM (∼29 molecules per RM).

#### 4.5.7 ^1^H-^1^H 2D NOESY NMR Spectroscopic Studies of UQ-2 in a *w*_*0*_ 12 AOT/Isooctane RM

2D NMR spectra were obtained using similar conditions used previously (Peters et al., 2016; Koehn et al., 2018b) using a 400 MHz Varian NMR at 26°C. A standard NOESY pulse sequence was used consisting of 256 transients with 16 scans in the f1 direction using a 200 ms mixing time, 45° pulse angle, and a relaxation delay of 1.5 s. The NMR instrument was locked onto 10% D_2_O signal. The resulting spectrum was processed using MestReNova NMR software version 10.0.1. (see Supplementary Material for details). The spectrum was referenced to the isooctane methyl peak at 0.904 ppm as previously reported (Samart et al., 2014; Koehn et al., 2018a). The 3D structure illustration within a RM was drawn using ChemBioD Ultra 12.0 and ChemBio3D Ultra 12.0 based on spectral parameters described under results.

### 4.6 Langmuir Monolayer Compression Isotherm Methods

#### 4.6.1 Instrument and Cleaning

All Langmuir monolayer studies were performed on a Kibron μTrough XS equipped with a Teflon ribbon barrier (hydrophobic) as described previously (Van Cleave et al., 2021). The trough bed was cleaned between runs by scrubbing three times with isopropanol, then scrubbing three times with absolute EtOH, and then rinsing with DDI H_2_O. The ribbon was cleaned by a rinse with isopropanol, a rinse with absolute EtOH, and then a rinse with DDI H_2_O.

#### 4.6.2 Preparation of the Subphase

The subphase consisted of approximately 50 ml of 20 mM sodium phosphate buffer (pH 7.40 ± 0.02). The pH was adjusted using 1.0 M HCl or NaOH. The subphase surface was cleaned with vacuum aspiration until the surface pressure remained at 0.0 ± 0.5 mN/m throughout a quick compression.

#### 4.6.3 Preparation of Lipid Solutions

Phospholipid solutions were prepared by dissolving powdered lipid (0.018 g DPPC, 0.017 g DPPE) into 25 ml of 9:1 chloroform/MeOH (v/v) to yield a 1 mM phospholipid stock solution. A 1 mM UQ-2 solution was prepared the same as the phospholipids, but with 0.0016 g UQ-2 dissolved into 5 ml of the chloroform/methanol solution. Stock solutions were stored at −20°C. Mixed monolayer were prepared immediately before experiments by adding appropriate amount of phospholipid stock and UQ-2 stock to a small glass vial and vortexing for ∼30 s. Mixed monolayers consisted of 25:72, 50:50, and 75:25 UQ-2:phospholipid (mol fraction).

#### 4.6.4 Formation and Compression of Monolayers

Monolayers consisted of pure DPPC, pure DPPE, pure UQ-2, or varying phospholipid:UQ-2 molar fractions (25:75, 50:50, 75:25). Films were prepared by adding 20 μl lipid stock solutions or mixtures (40 µl were used for UQ-2 and 75:25 UQ-2:phospholipid to obtain full compression) drop-wise to the surface of the subphase and were equilibrated for 15 min. Monolayers were compressed at a speed of 10 mm/min (5 mm/min from two sides). Surface pressure measurements were made via a modified Wilhelmy plate method where a wire probe was used instead of a plate. Surface pressure was calculated from surface tension with Eq. 1, where π is surface pressure (mN/m), $\gamma_{o}$ is the surface tension of the subphase (72.8 mN/m), and γ is the surface tension after the addition of the monolayer.

$$\pi =\gamma_{o}-\gamma .$$ (1)

The averages of triplicate isotherms were worked up in Excel. The averages were then normalized to the amount of phospholipid according to Eq. 2, where A_N_ is the normalized area per phospholipid (Å^2^), A is the experimental area per molecule (Å^2^) (Van Cleave et al., 2021), and x is the mol fraction of phospholipid (0.25, 0.5, or 0.75). This method of analysis was developed from a previous study (Quinn and Esfahani, 1980).

$$A_{N}=A\left(x^{-1}\right).$$ (2)

Normalized isotherms were plotted with Origin 2021. Reported error bars are the standard deviations of the experimental area.

## Data Availability

The original contributions presented in the study are included in the article/Supplementary Material, further inquiries can be directed to the corresponding author.
