# Supplementary material for: Electron Transport Lipids Fold Within Membrane-Like Interfaces
Source: Front Chem. 2022 Mar 8;10:827530. doi: 10.3389/fchem.2022.827530 (PMC8957872; doi:10.3389/fchem.2022.827530)
Supplement: Supplementary file 1 [file DataSheet1.pdf]

Electronic Supplementary Materials

Table of Contents:

I NMR spectra for synthesized compounds

- **Fig. S1.** ^{1}H NMR (400 MHz) spectrum of 2,3,4-trimethoxy-6-methylbenzaldehyde (2) in $CDCl_3$………3
- **Fig. S2.** ^{13}C NMR (101 MHz) spectrum of 2,3,4-trimethoxy-6-methylbenzaldehyde (2) in $CDCl_3$……..4
- **Fig. S3.** ^{1}H NMR (400 MHz) spectrum of 2,3,4-trimethoxy-6-methylphenol (3) in $CDCl_3$ …..…….......5
- **Fig. S4.** ^{13}C NMR (101 MHz) spectrum of 2,3,4-trimethoxy-6-methylphenol (3) in $CDCl_3$ ……………..6
- **Fig. S5.** ^{1}H NMR (400 MHz) spectrum of geranyl 2,3,4-trimethoxy-6-methylphenyl ether (6) in $CDCl_3$…7
- **Fig. S6.** ^{13}C NMR (101 MHz) spectrum of geranyl 2,3,4-trimethoxy-6-methylphenyl ether (6) in $CDCl_3$..8
- **Fig. S7.** ^{1}H NMR (400 MHz) spectrum of 5-geranyl-2,3,4-trimethoxy-6-methyl-phenol (7) in $CDCl_3$……9
- **Fig. S8.** ^{13}C NMR (101 MHz) spectrum of 5-geranyl-2,3,4-trimethoxy-6-methyl-phenol (7) in $CDCl_3$…10
- **Fig. S9.** ^{1}H NMR (400 MHz) spectrum of ubiquinone-2 (8) in $CDCl_3$ ……………………….……………11
- **Fig. S10.** ^{13}C NMR (101 MHz) spectrum of ubiquinone-2 (8) in $CDCl_3$ …………………………………..12
- **Fig. S11**. ^{1}H-^{1}H 2D gCOSY NMR (400 MHz) spectrum of ubiquinone-2 (8) in $CDCl_3$ …….……………13
- **Fig. S12.** ^{1}H-^{13}C 2D HSQC NMR (400 MHz) spectrum of ubiquinone-2 (8) in $CDCl_3$……….............14

II NMR spectra for UQ-2

- **Fig. S13.** ^{1}H-^{1}H 2D ROESY NMR (400 MHz) spectrum of UQ-2 in d_6-DMSO……………………………15
- **Fig. S14.** ^{1}H-^{1}H 2D gCOSY NMR (400 MHz) spectrum of UQ-2 in d_6-DMSO……………….……………16
- **Fig. S15.** ^{1}H-^{1}H 2D gCOSY NMR (400 MHz) spectrum of UQ-2 in d_3-acetonitrile………..…………17
- **Fig. S16.** ^{1}H-^{1}H 2D NOESY NMR (400 MHz) spectrum of UQ-2 in d_3-acetonitrile……….……………18
- **Fig. S17.** ^{1}H-^{1}H 2D ROESY NMR (400 MHz) spectrum of UQ-2 in d_3-acetonitrile ..…..………………19
- **Fig. S18.** ^{1}H-^{1}H 2D gCOSY NMR (400 MHz) spectrum of UQ-2 in C_6D_6…………………….…..………20
- **Fig. S19.** (A) ^{1}H-^{1}H 2D Full NOESY NMR (400 MHz) spectrum of UQ-2 in C_6D_6. (B) ^{1}H-^{1}H 2D Partial NOESY NMR (400 MHz) spectrum of UQ-2 in C_6D_6. ……………………………….…………………..21
- **Fig. S20.** ^{1}H-^{1}H 2D ROESY NMR (400 MHz) spectrum of UQ-2 in C_6D_6. ………………….…………..22
- **Fig. S21.** ^{1}H-^{1}H 2D gCOSY NMR (400 MHz) spectrum of UQ-2 in d_5-pyridine. …..…..……………......23
- **Fig. S22.** (A) ^{1}H-^{1}H 2D Full NOESY NMR (400 MHz) spectrum of UQ-2 in d_5-pyridine. (B) ^{1}H-^{1}H 2D Full NOESY NMR (400 MHz) spectrum of UQ-2 in d_5-pyridine...………………...……..……...24
- **Fig. S23.** ^{1}H-^{1}H 2D ROESY NMR (400 MHz) spectrum of UQ-2 in d_5-5yridine…………………………25
- **Fig. S24.** ^{1}H-^{1}H 1D NOESY NMR (400 MHz) spectrum of UQ-2 in w_0 12 reverse micelles with irradiated H_A and H_B protons …………………………………………………………………………………26

III Molecular Mechanics: conformational analysis of UQ-2 and MK-2 and proton distances

- **Fig. S25.** Comparison of superimposed 3D conformations of UQ-2 based on the 2D NMR data in d_3-acetonitrile / d_6-DMSO and d_6-benzene / d_5-pyridine……………………………………..…………..27
- **Table S1.** Comparison of interproton distances of UQ-2 and MK-2 in d_6-DMSO and d_5-pyridine……...28
- **Table S2.** Comparison of interproton distances of UQ-2 and MK-2 w_0 12 reverse micelles……………28

IV Langmuir Monolayers studies of UQ-2

- **Methods:** Compression Modulus Analysis of Langmuir Monolayers…………………….……………...29
- **Fig. S26.** The compression moduli of normalized UQ-2 mixed monolayers of (A) DPPC or (B) DPPE..29
- **Table S3.** Percent difference between the area of mixed DPPC:UQ-2 monolayers and the DPPC control…………………………………………………………………………………….……………29
- **Table S4.** Percent difference between the area of mixed DPPE:UQ-2 monolayers and the DPPE control…………………..………………………………………………………………..……...30

V Dynamic Light Scattering data for UQ-2

- RM Sample Preparation for Dynamic Light Scattering (DLS) Studies ………..……………………….31
- **Table S5.** Dynamic Light Scattering measurements on UBQ-2 in 0.1 M AOT/isooctane RMs………..31
- DLS Measurements…………………………..………………………………………..……..…...31
- DLS measurements of UBQ-2 containing AOT/isooctane RMs……………………….…………….…31

I NMR spectra for synthesized compounds

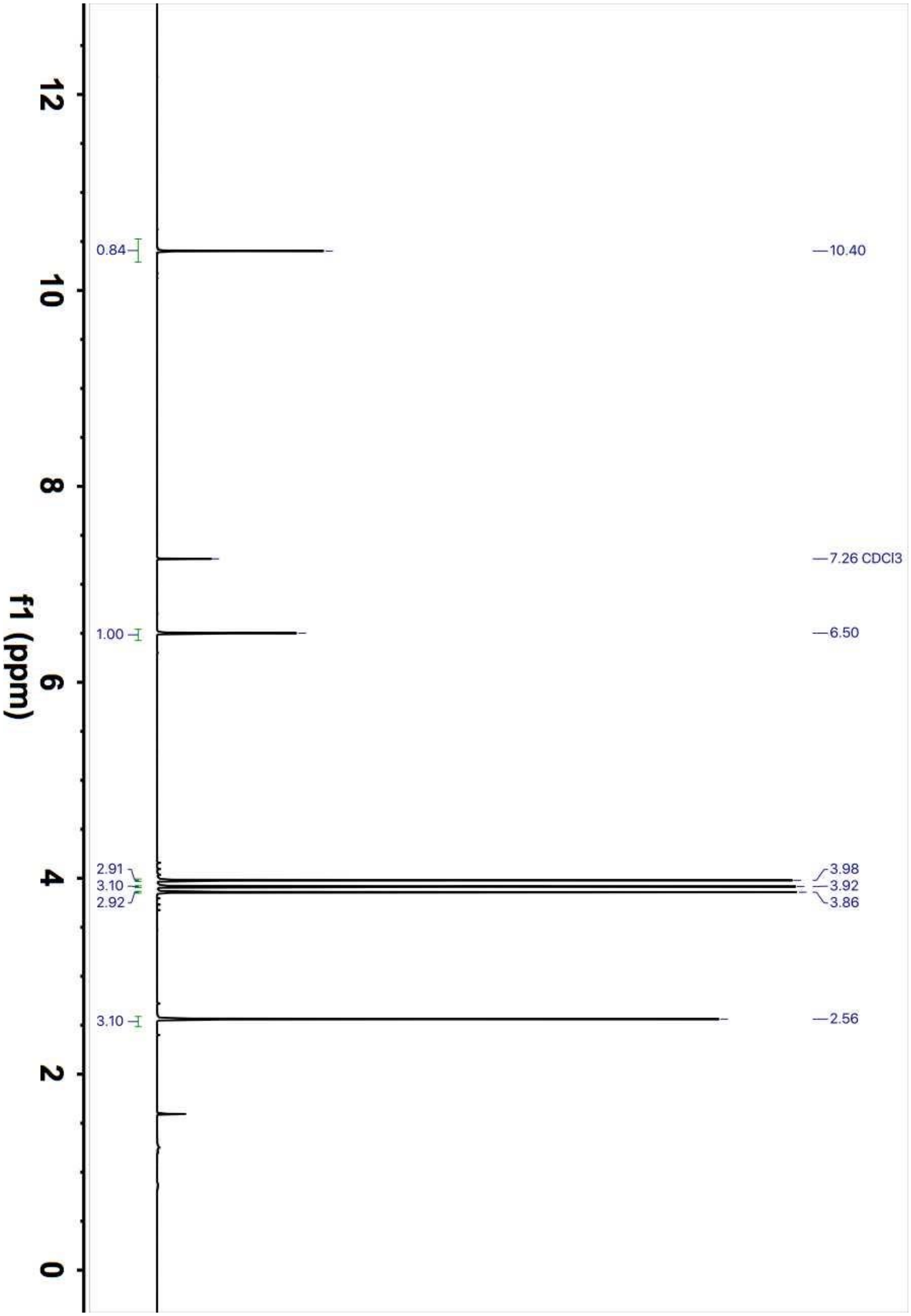

Fig. S1. ^{1}H NMR (400 MHz) spectrum of 2,3,4-trimethoxy-6-methylbenzaldehyde (2) in $CDCl_3$

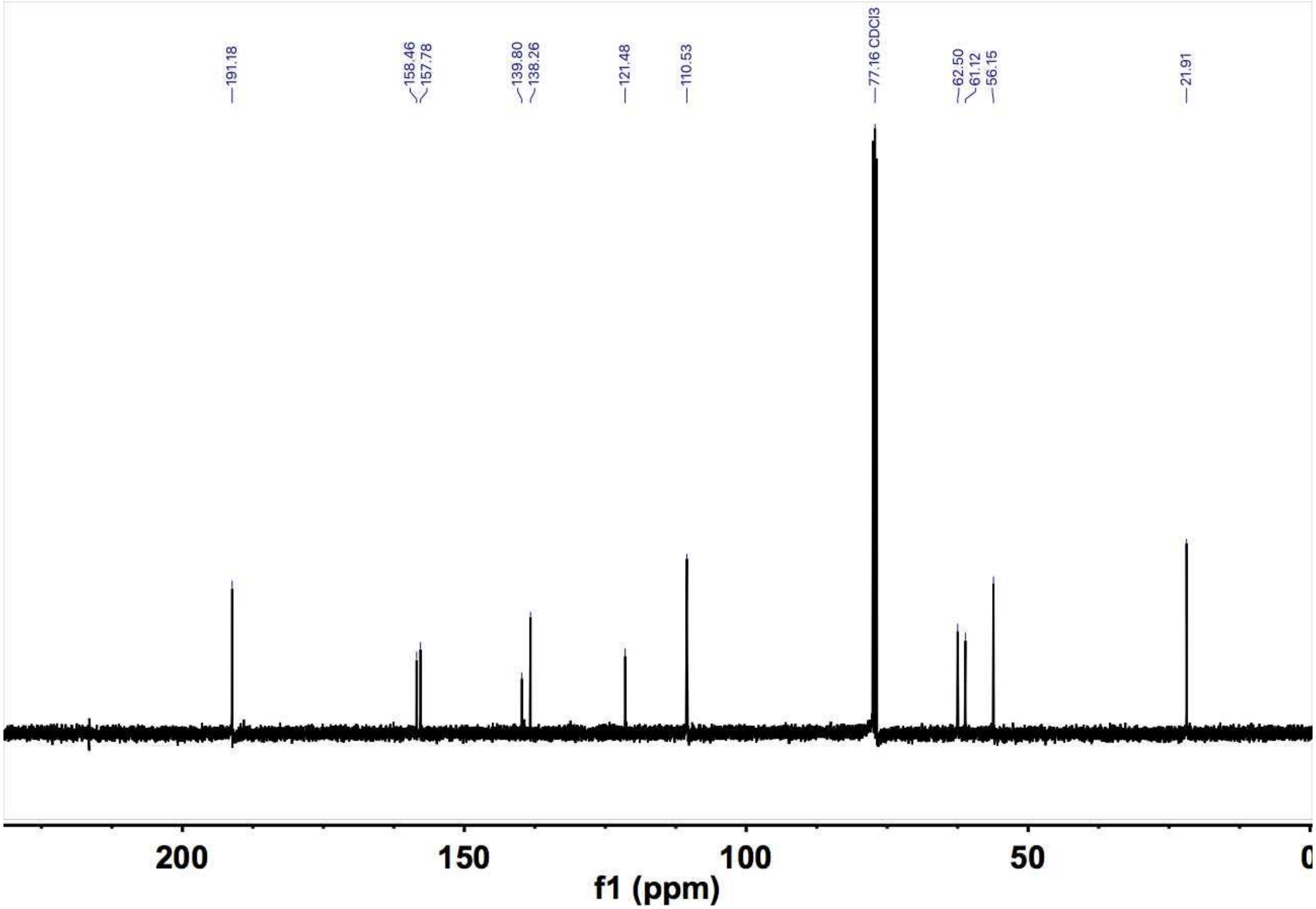

Fig. S2. ^{13}C NMR (101 MHz) spectrum of 2,3,4-trimethoxy-6-methylbenzaldehyde (2) in $CDCl_3$

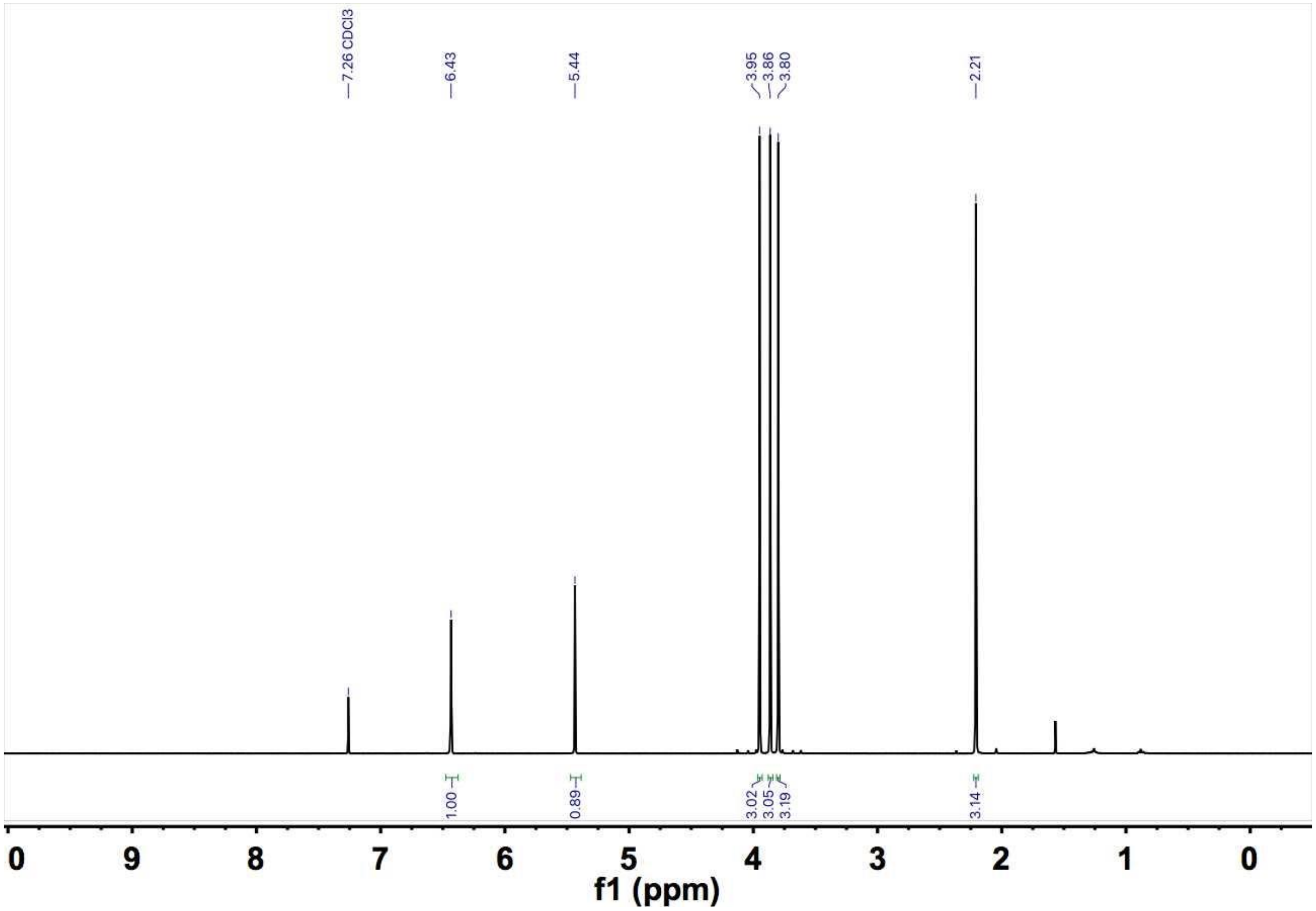

Fig. S3. ^{1}H NMR (400 MHz) spectrum of 2,3,4-trimethoxy-6-methylphenol (3) in $CDCl_3$

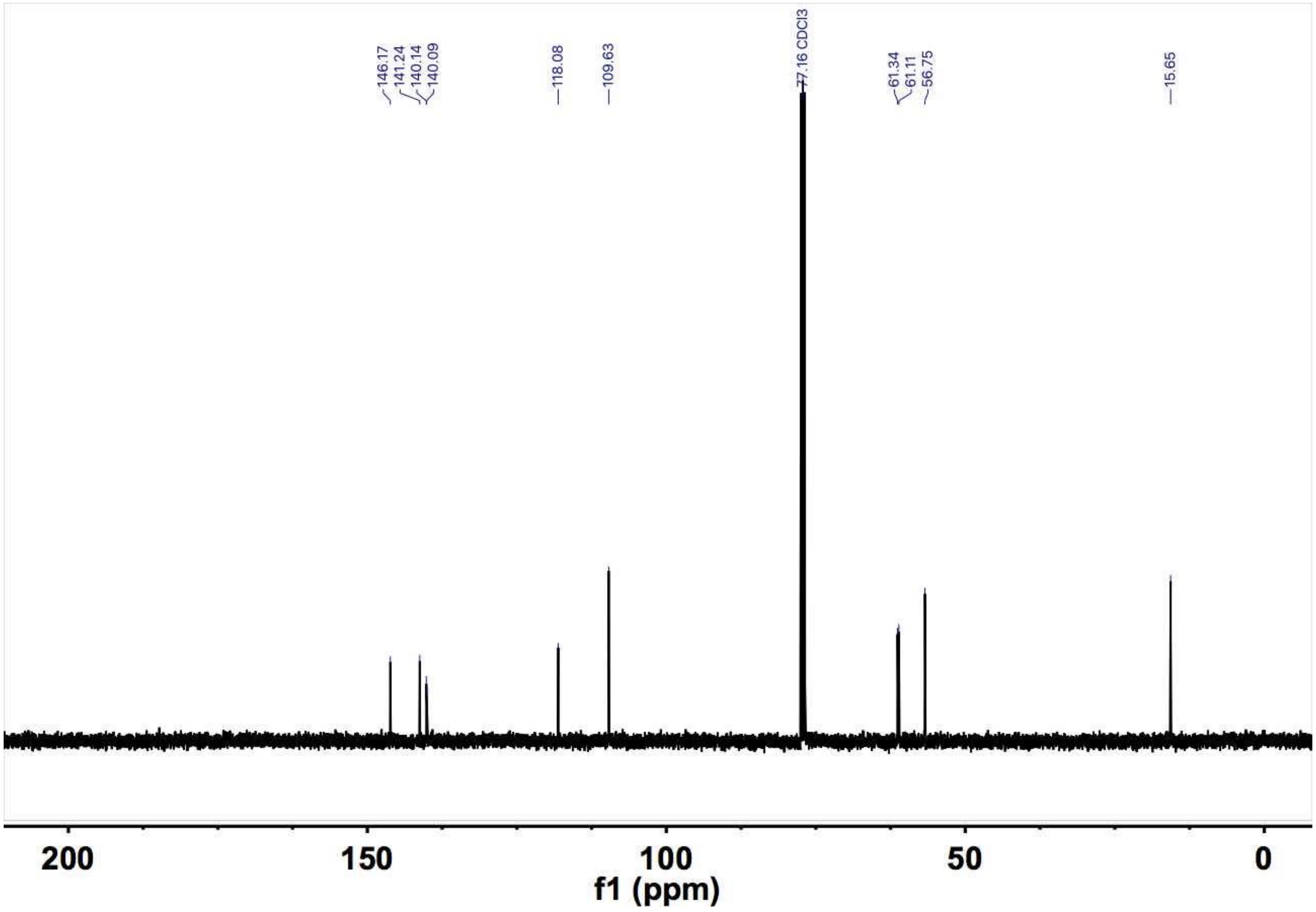

Fig. S4. ^{13}C NMR (101 MHz) spectrum of 2,3,4-trimethoxy-6-methylphenol (3) in $CDCl_3$

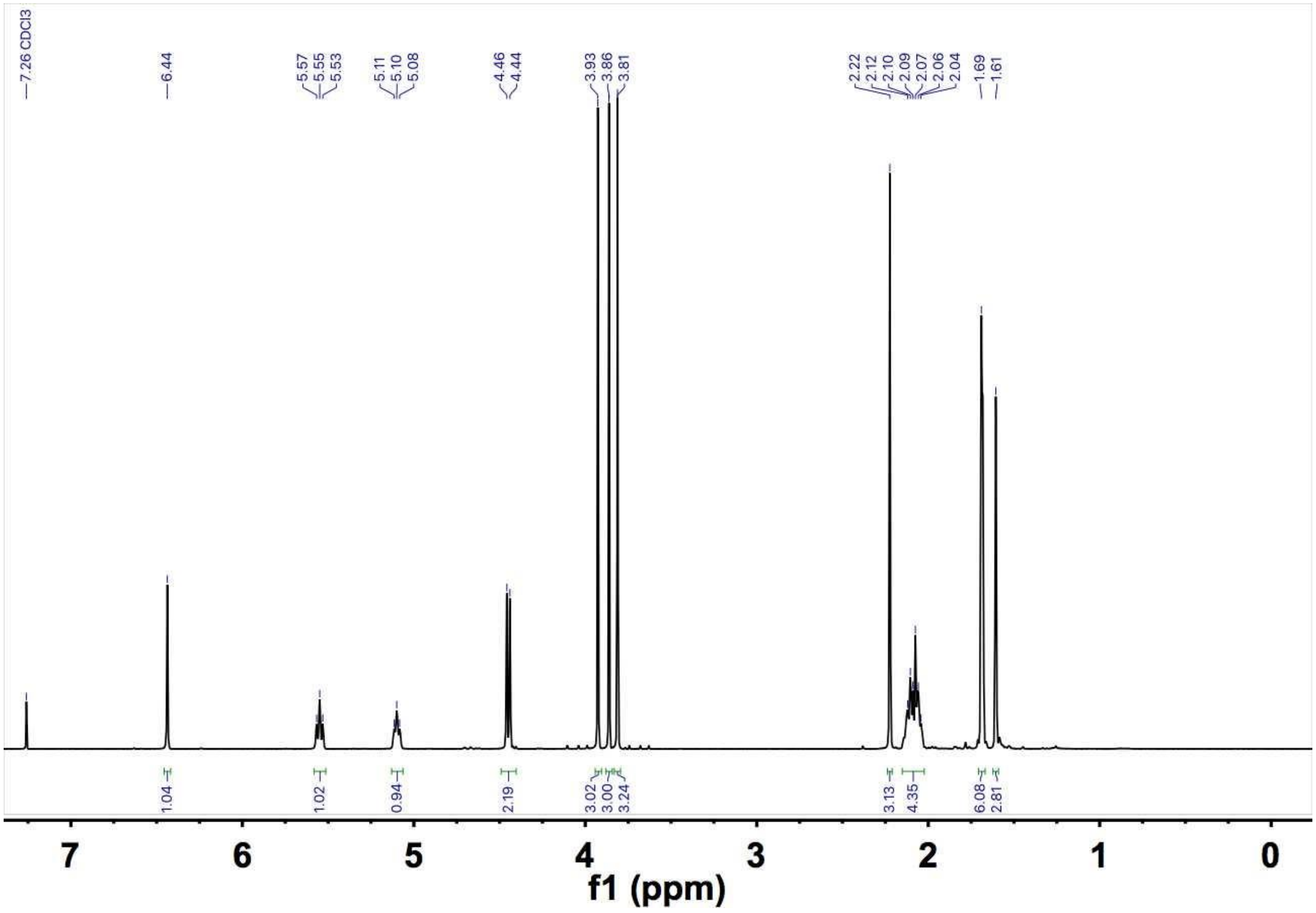

Fig. S5. ^{1}H NMR (400 MHz) spectrum of geranyl 2,3,4-trimethoxy-6-methylphenyl ether (6) in $CDCl_3$

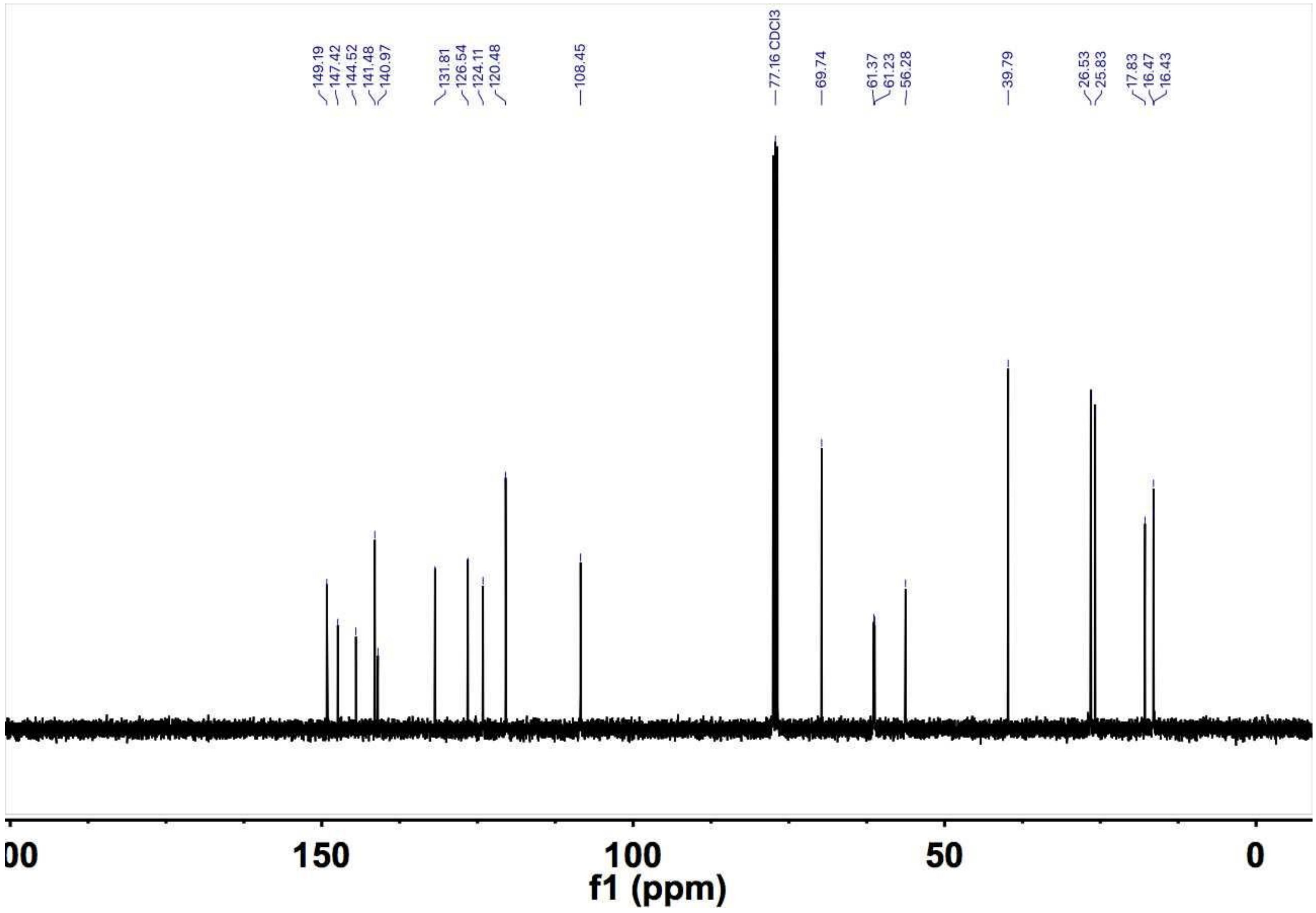

Fig. S6. ^{13}C NMR (101 MHz) spectrum of geranyl 2,3,4-trimethoxy-6-methylphenyl ether (6) in $CDCl_3$

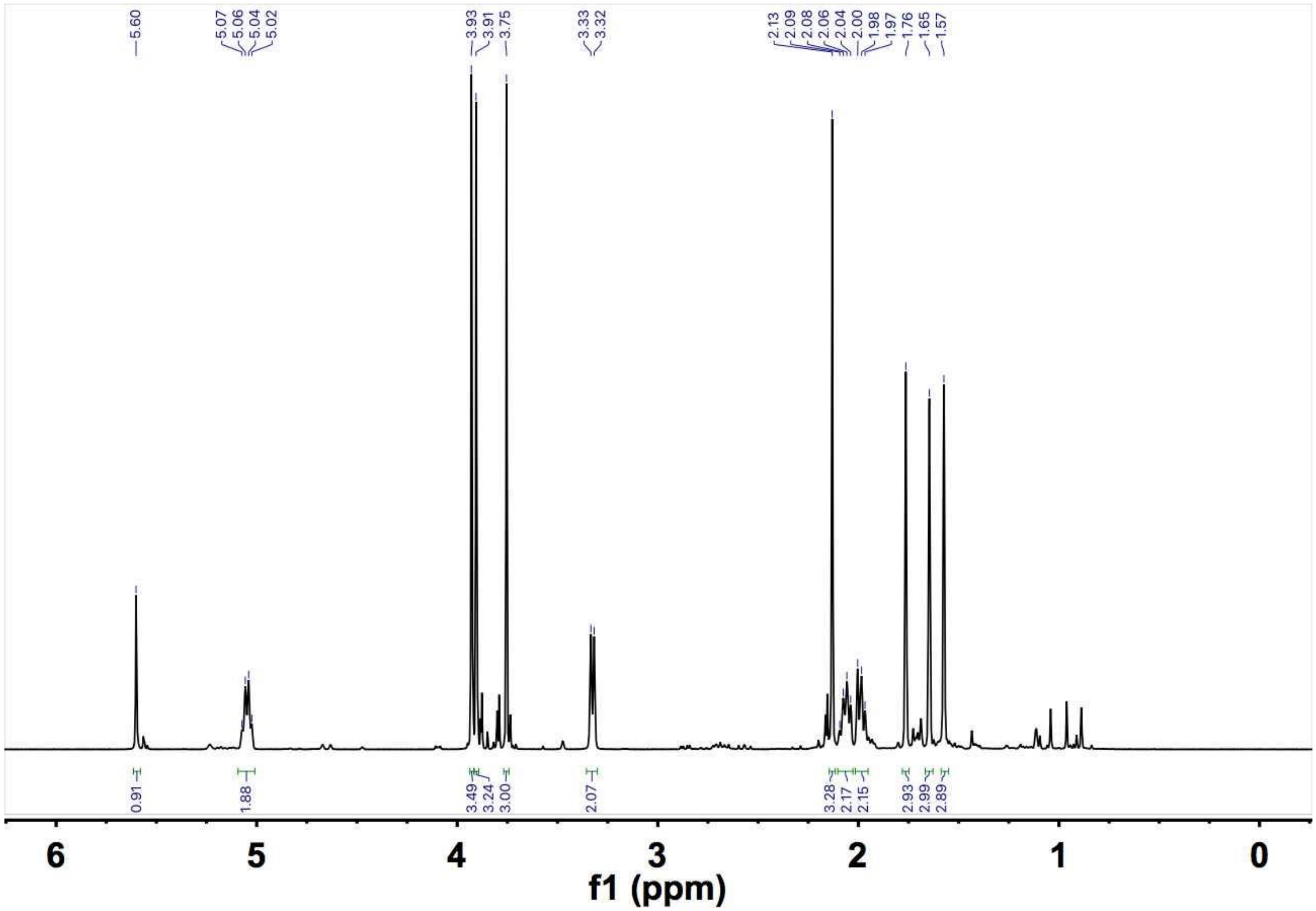

Fig. S7. ^{1}H NMR (400 MHz) spectrum of 5-geranyl-2,3,4-trimethoxy-6-methyl-phenol (7) in $CDCl_3$

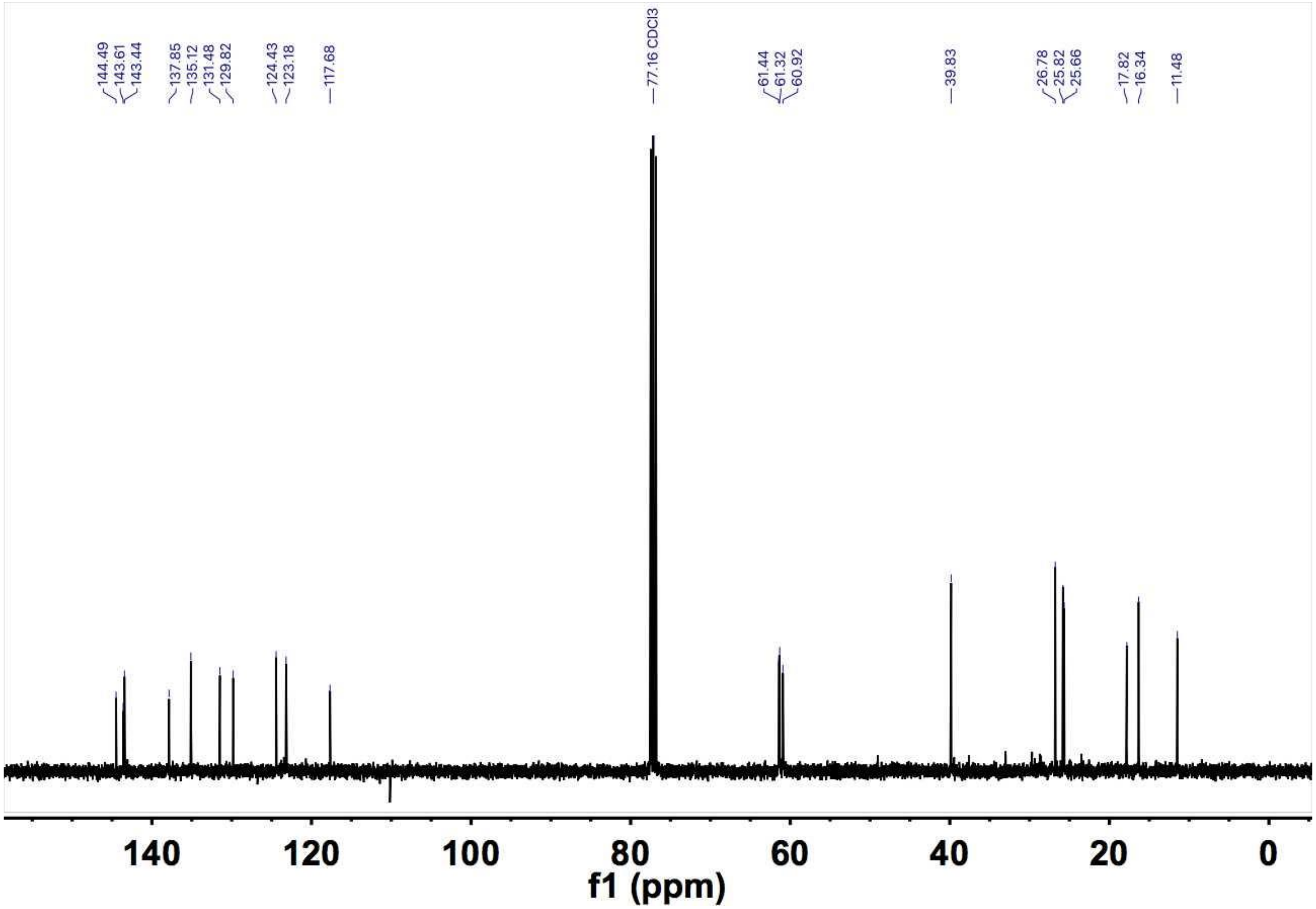

Fig. S8. ^{13}C NMR (101 MHz) spectrum of 5-geranyl-2,3,4-trimethoxy-6-methyl-phenol (7) in $CDCl_3$

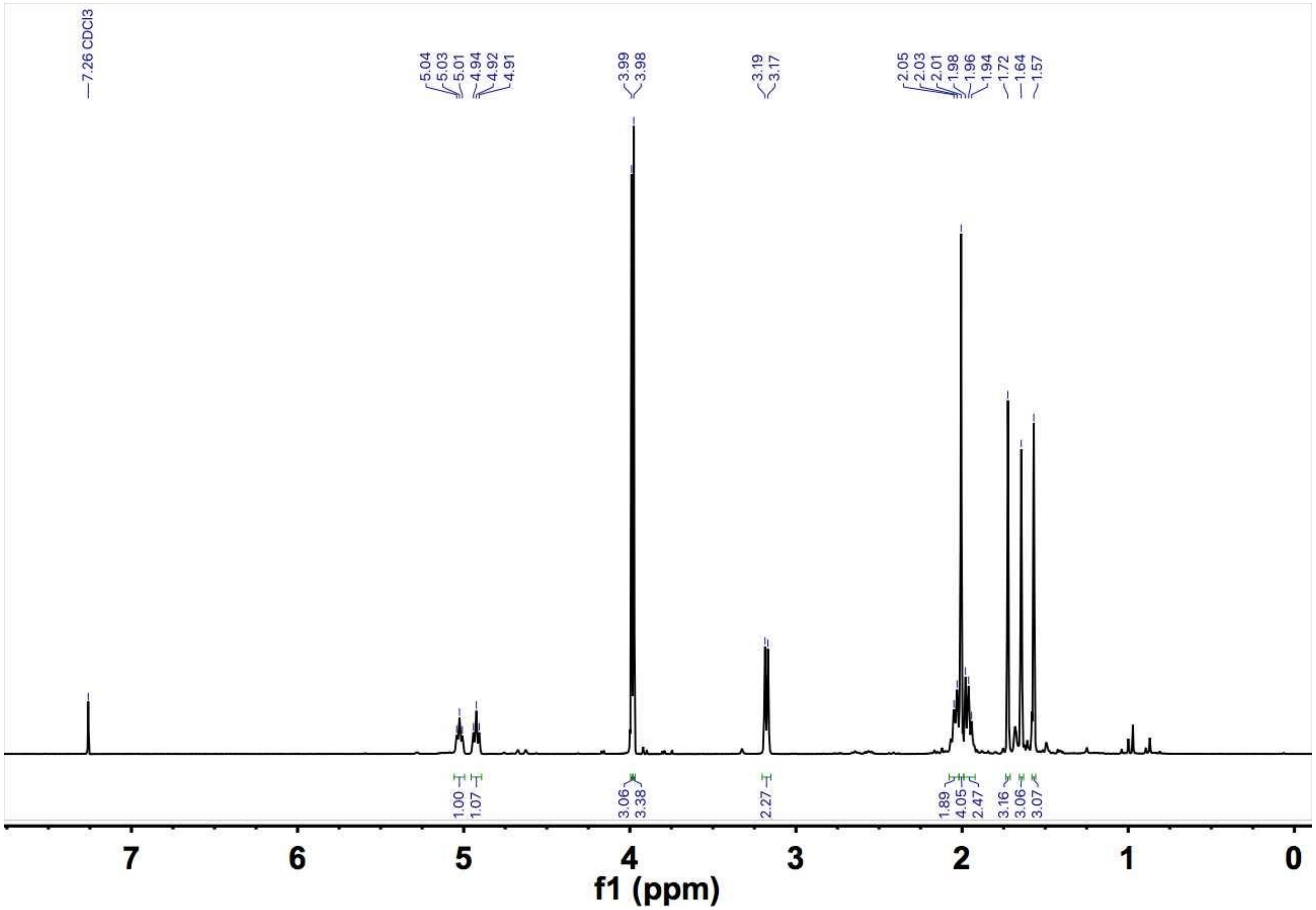

Fig. S9. ^{1}H NMR (400 MHz) spectrum of ubiquinone-2 (8) in $CDCl_3$

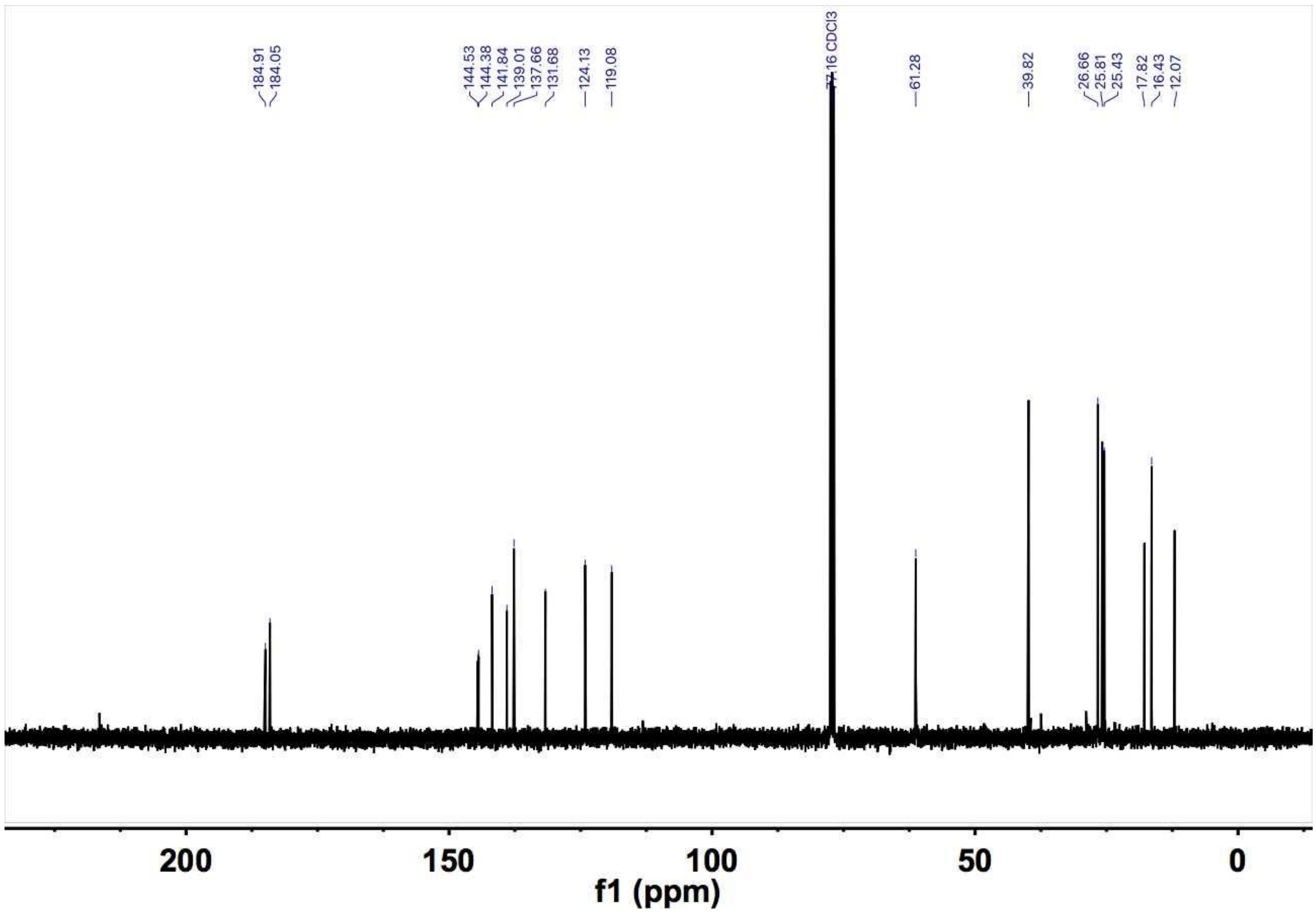

Fig. S10. ^{13}C NMR (101 MHz) spectrum of ubiquinone-2 (8) in $CDCl_3$

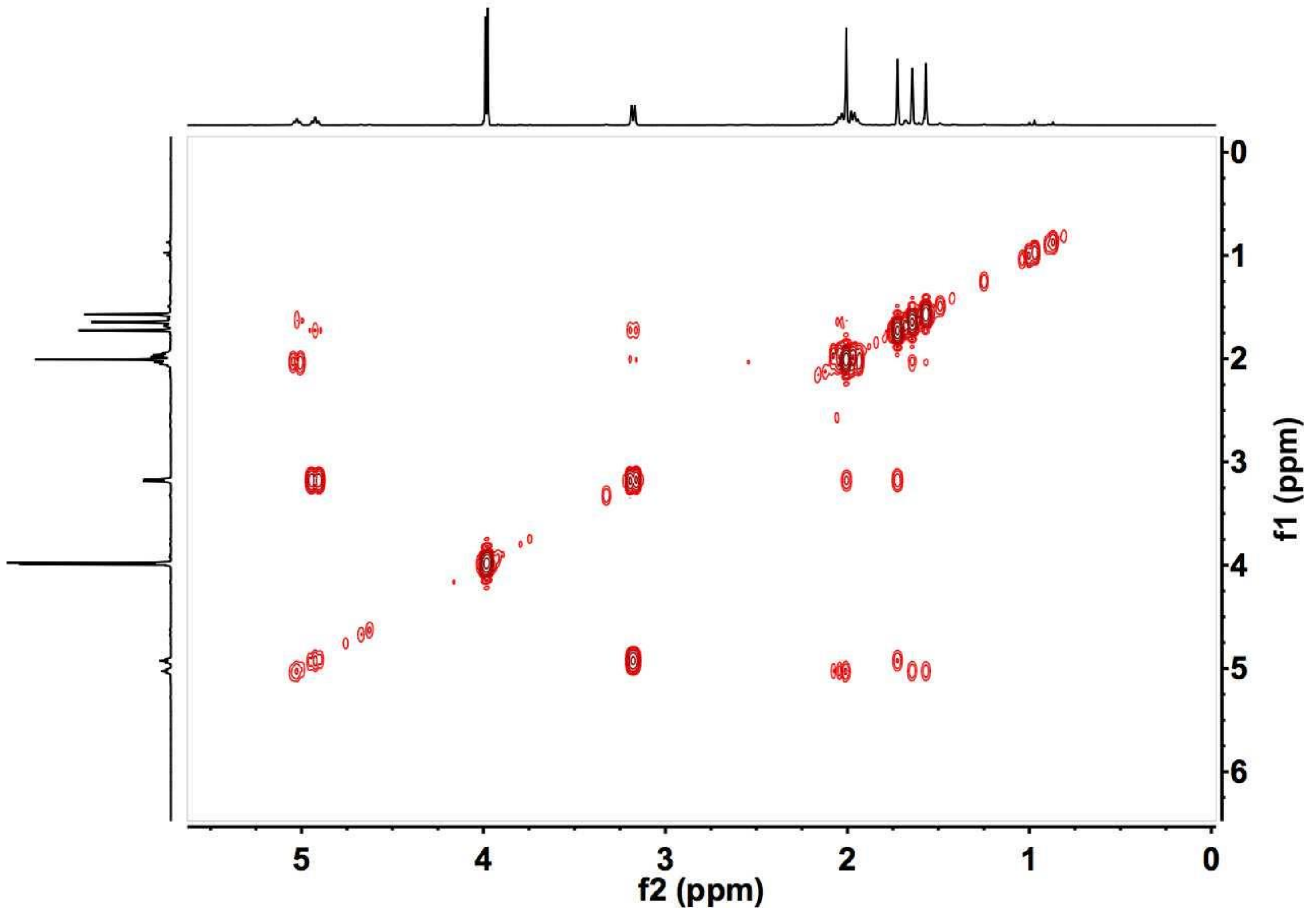

Fig. S11. ^{1}H-^{1}H 2D gCOSY NMR (400 MHz) spectrum of ubiquinone-2 (8) in $CDCl_3$

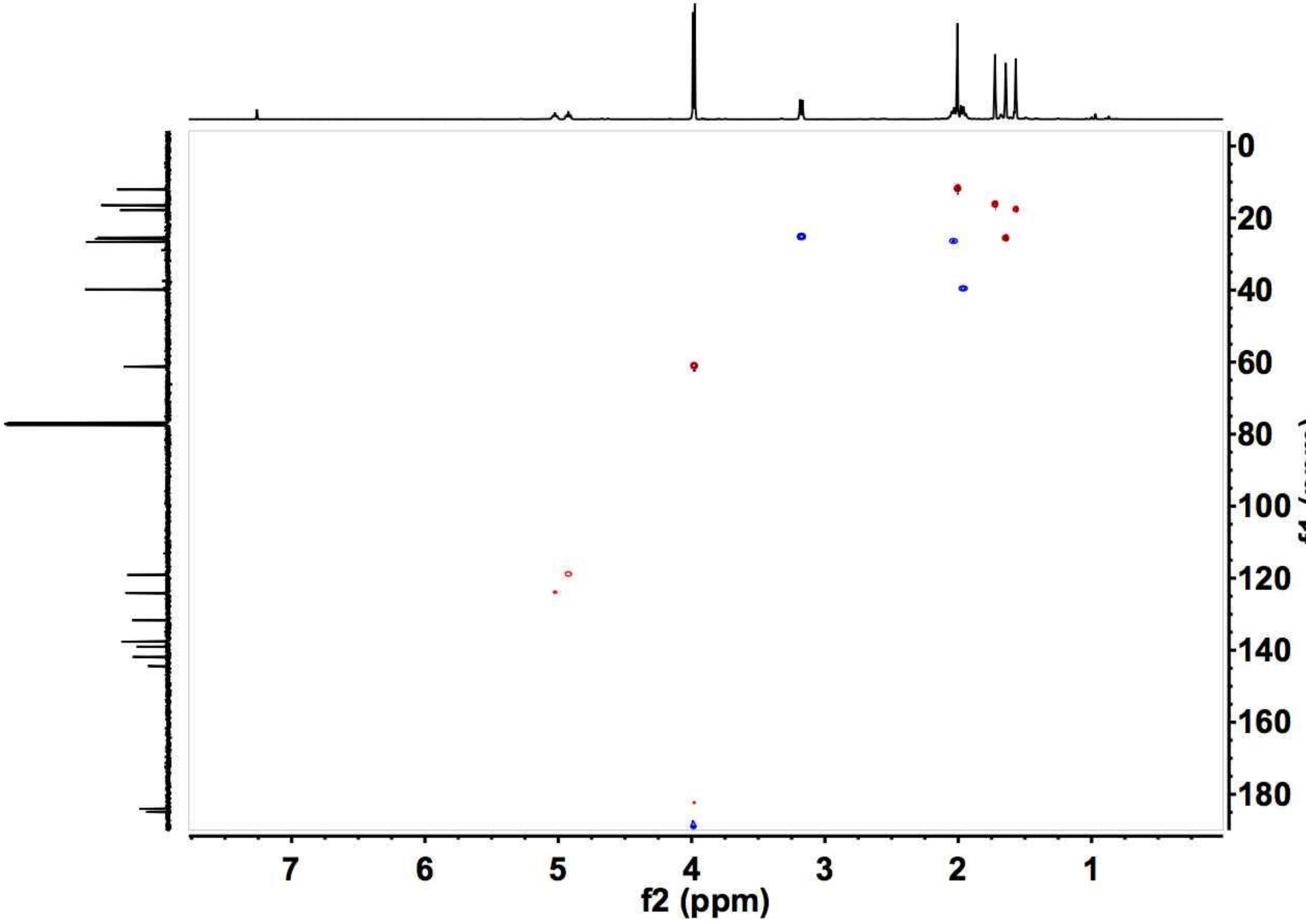

Fig. S12. ^{1}H-^{13}C 2D HSQC NMR (400 MHz / 101 MHz) spectrum of ubiquinone-2 (8) in $CDCl_3$

II NMR spectra for UQ-2

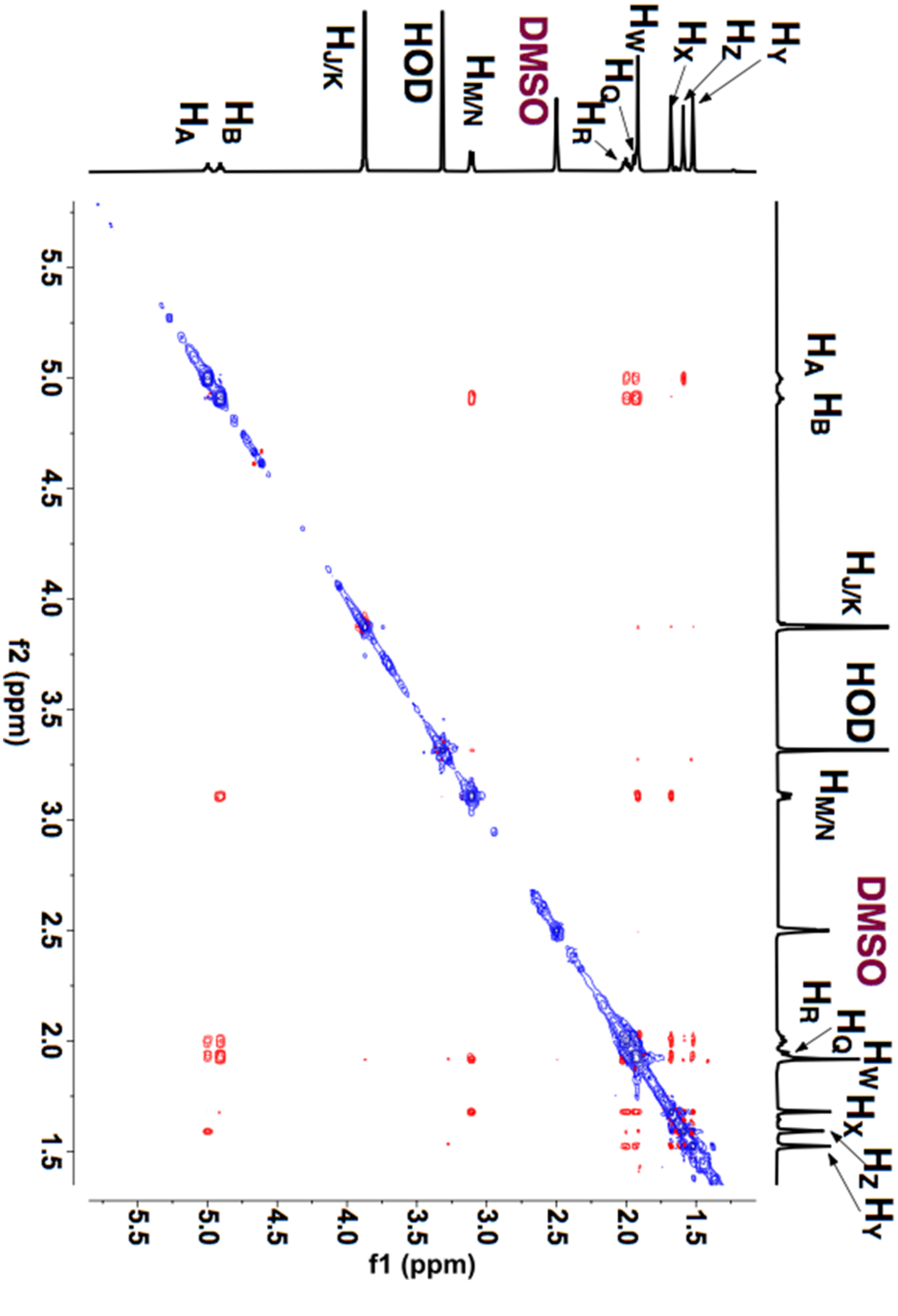

Fig. S13. ^{1}H-^{1}H 2D ROESY NMR (400 MHz) spectrum of 20.0 mM UQ-2 at 25 °C in d_6-DMSO. A standard ROESYAD pulse sequence was used consisting of 256 transients with 16 scans in the f1 domain using a 400 ms mixing time and 2.0 sec. relaxation delay per t1 increment.

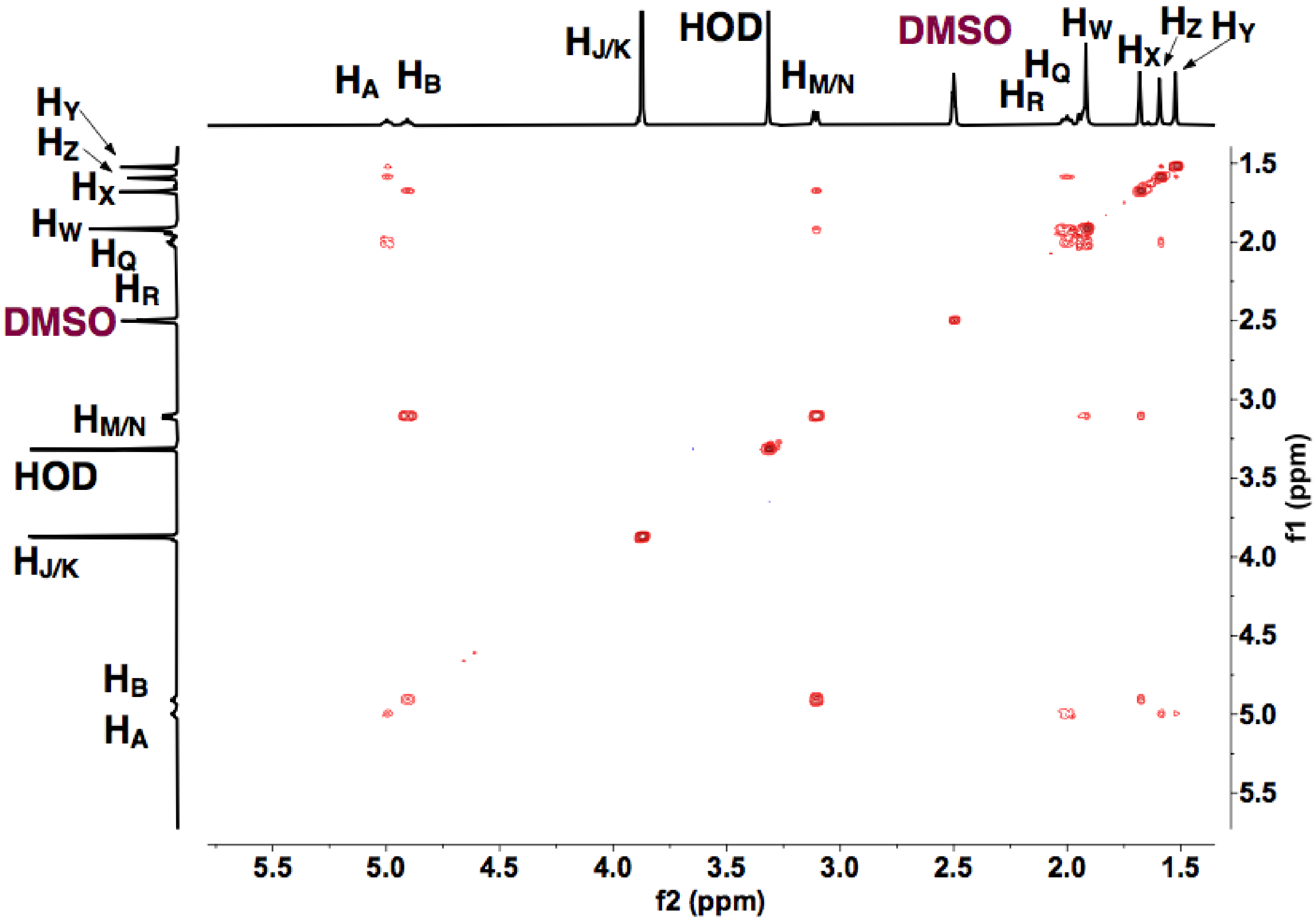

Fig. S14. ^1H-^1H 2D gCOSY NMR (400 MHz) spectrum of 20.0 mM UQ-2 in d_6-DMSO at 25 °C. A standard gCOSY pulse sequence was used consisting of 200 t1 increments with 8 scans per t1 increment.

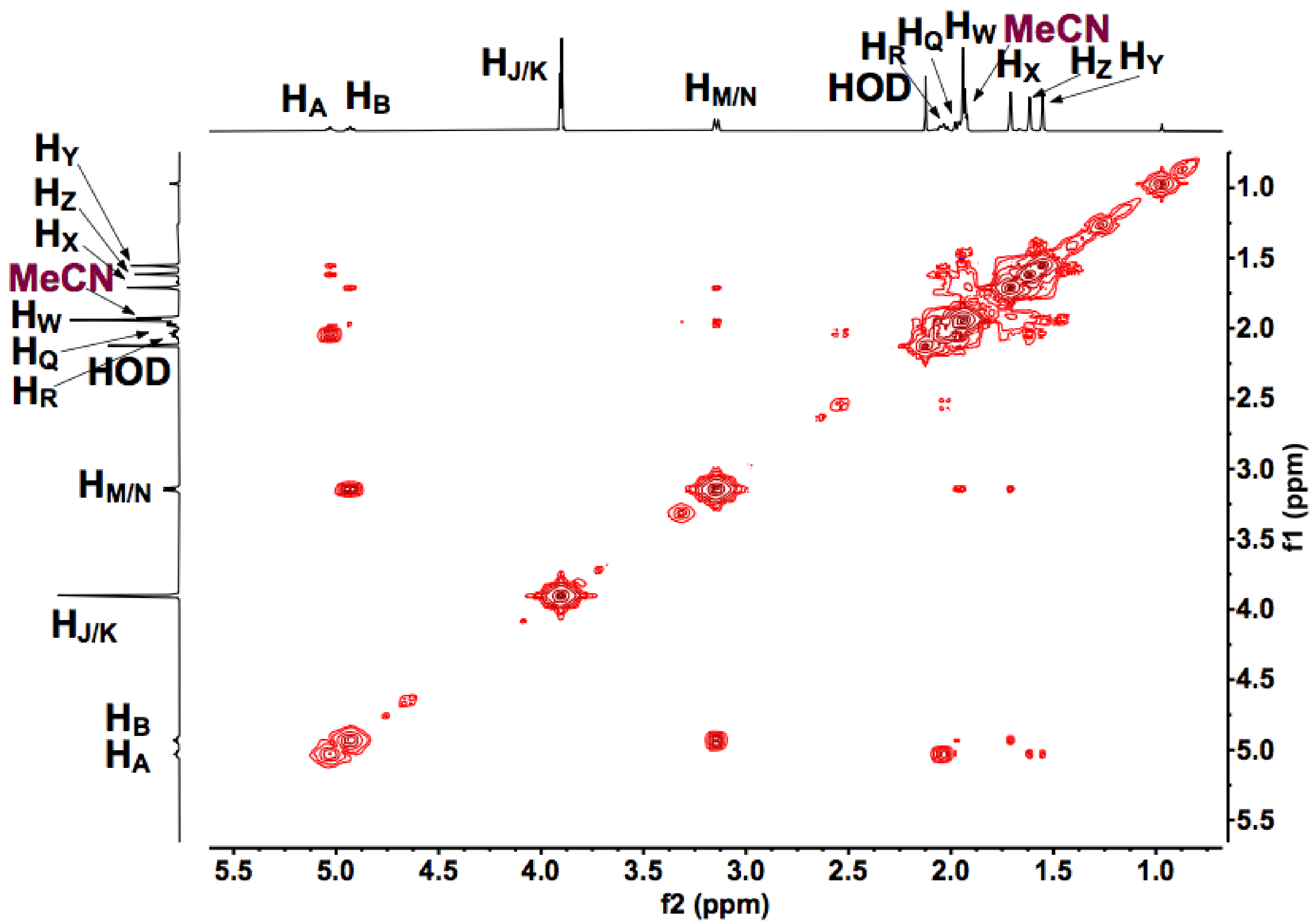

Fig. S15. ^{1}H-^{1}H 2D gCOSY NMR (400 MHz) spectrum of 20.0 mM UQ-2 in d_3-acetonitrile at 25 °C. A standard gCOSY pulse sequence was used consisting of 200 t1 increments with 8 scans per t1 increment.

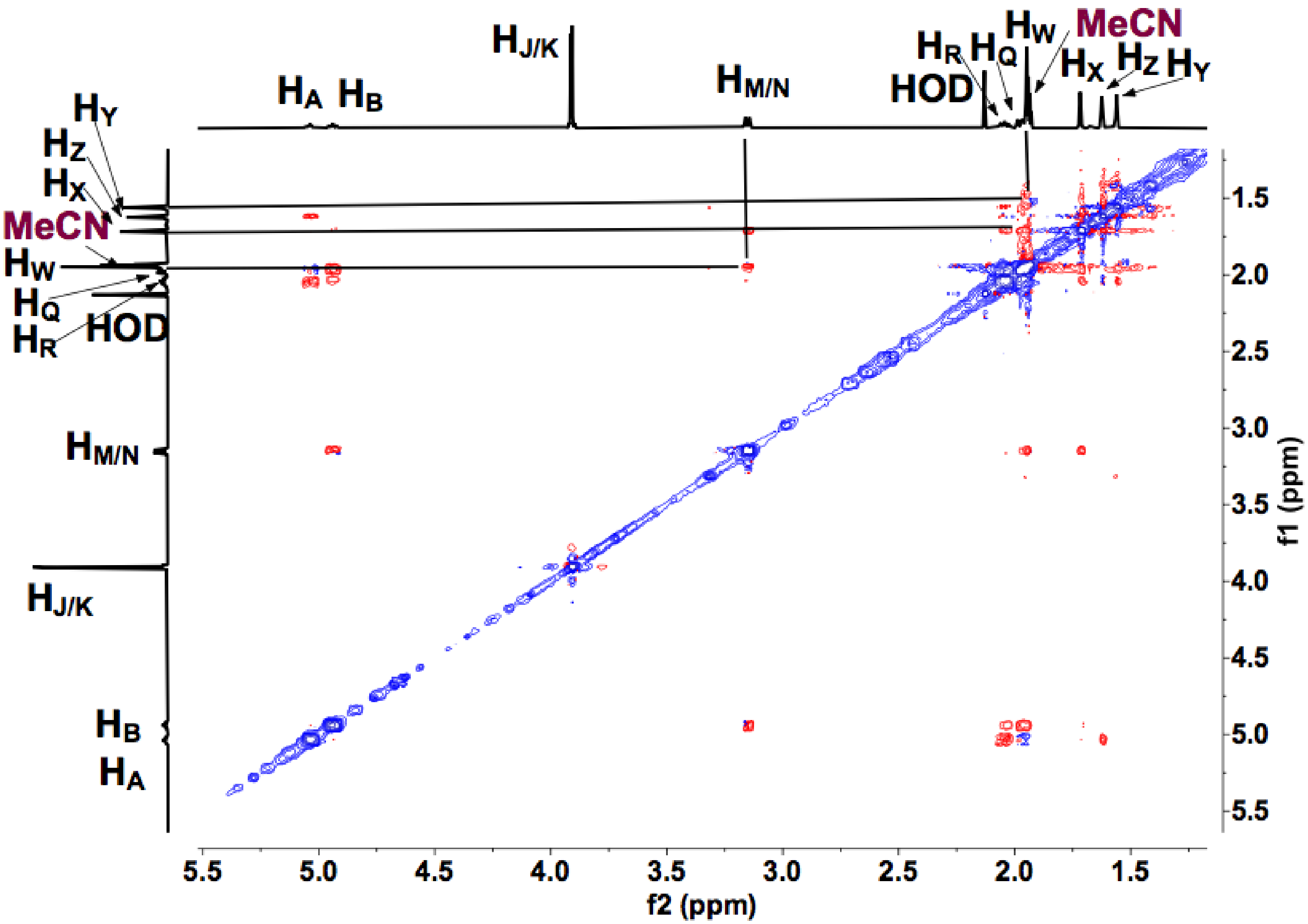

Fig. S16. ^{1}H-^{1}H 2D NOESY NMR (400 MHz) spectrum of 20.0 mM UQ-2 at 25 °C in d_3-acetonitrile. A standard ROESYAD pulse sequence was used consisting of 256 transients with 16 scans in the f1 domain using a 400 ms mixing time and 2.0 sec. relaxation delay per t1 increment.

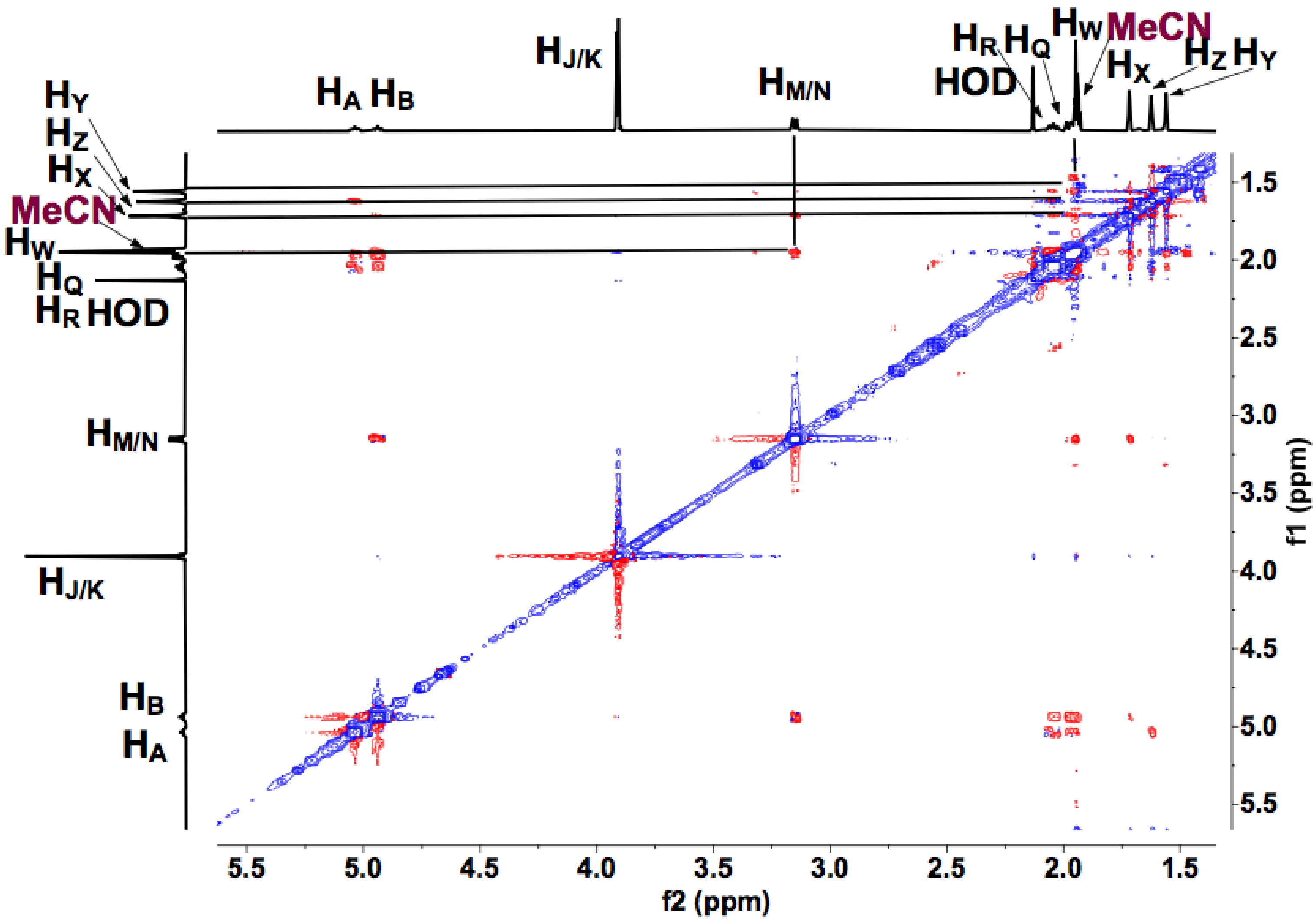

Fig. S17. ^{1}H-^{1}H 2D ROESY NMR (400 MHz) spectrum of 20.0 mM UQ-2 at 25 °C in d_3-acetonitrile. A standard ROESYAD pulse sequence was used consisting of 256 transients with 16 scans in the f1 domain using a 400 ms mixing time and 2.0 sec. relaxation delay per t1 increment.

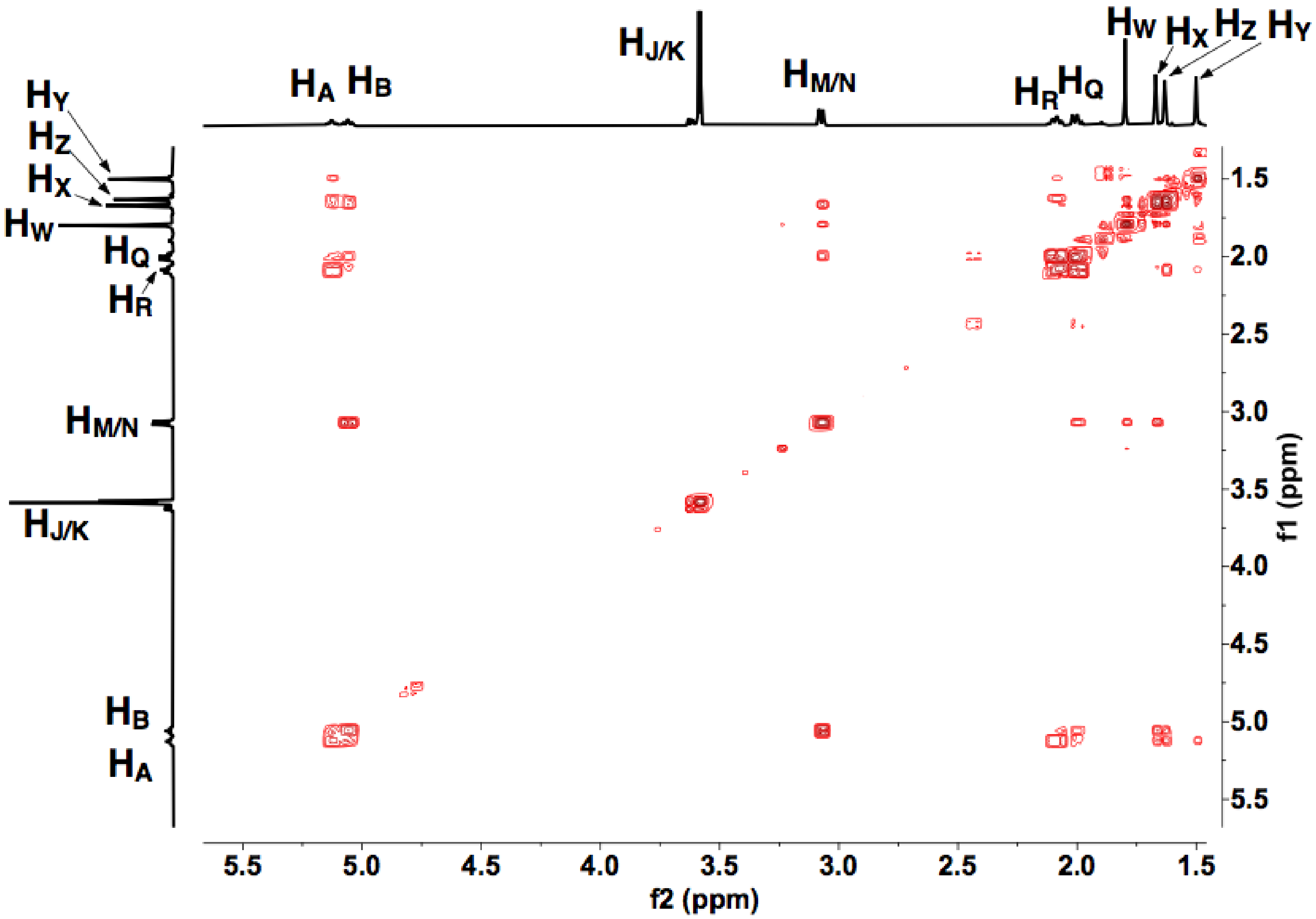

Fig. S18. ^1H-^1H 2D gCOSY NMR (400 MHz) spectrum of 20.0 mM UQ-2 in C_6D_6 at 25 °C. A standard gCOSY pulse sequence was used consisting of 200 t1 increments with 8 scans per t1 increment.

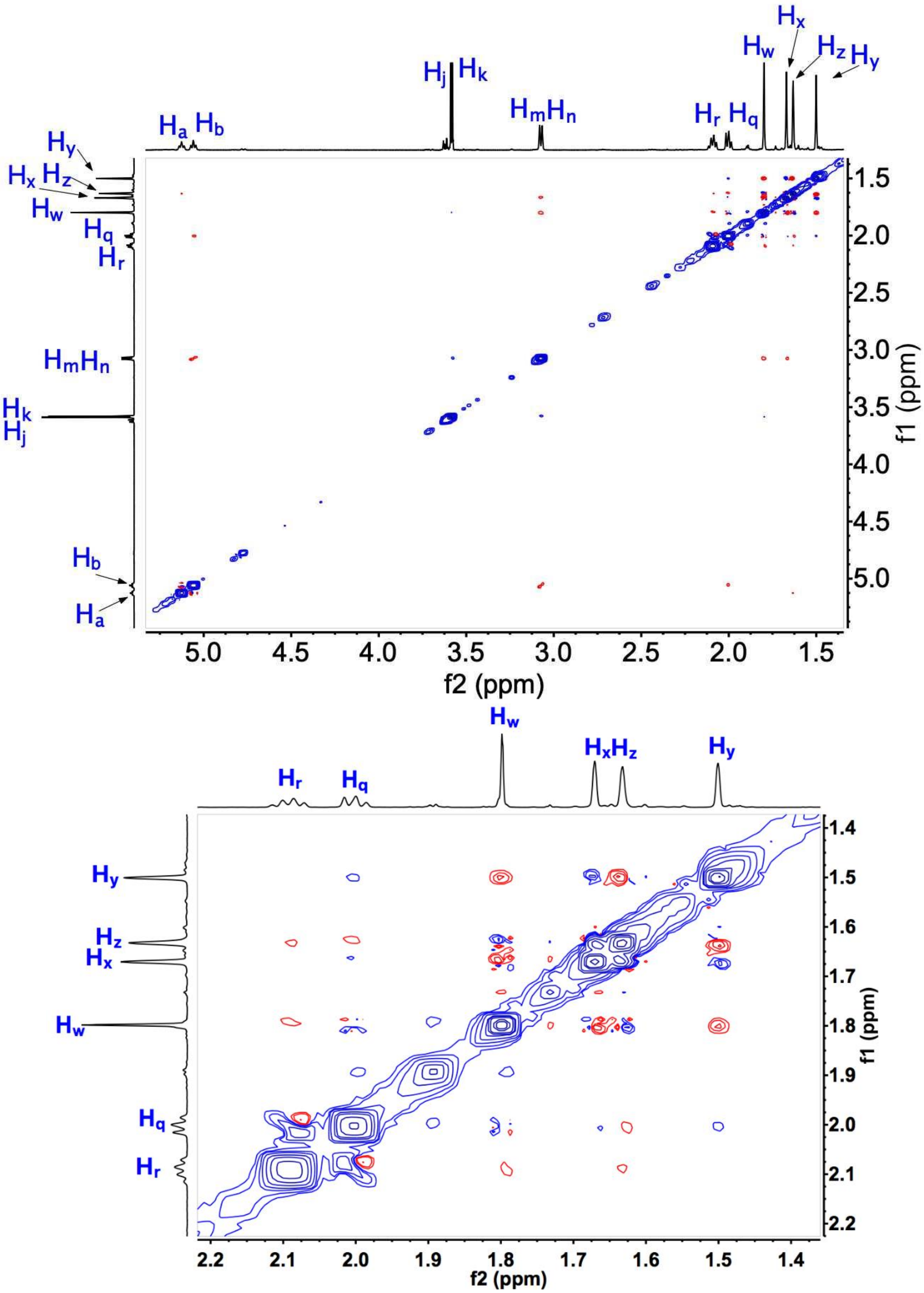

Figure S19. (A) ^{1}H-^{1}H 2D Full NOESY and (**B)** ^{1}H-^{1}H 2D Partial NOESY NMR (400 MHz) spectra of 20 mM UQ-2 at 25 °C in C_6D_6. A standard NOESY pulse sequence was used consisting of 256 transients with 16 scans in the f1 domain using a 500 ms mixing time and 1.5 sec relaxation delay.

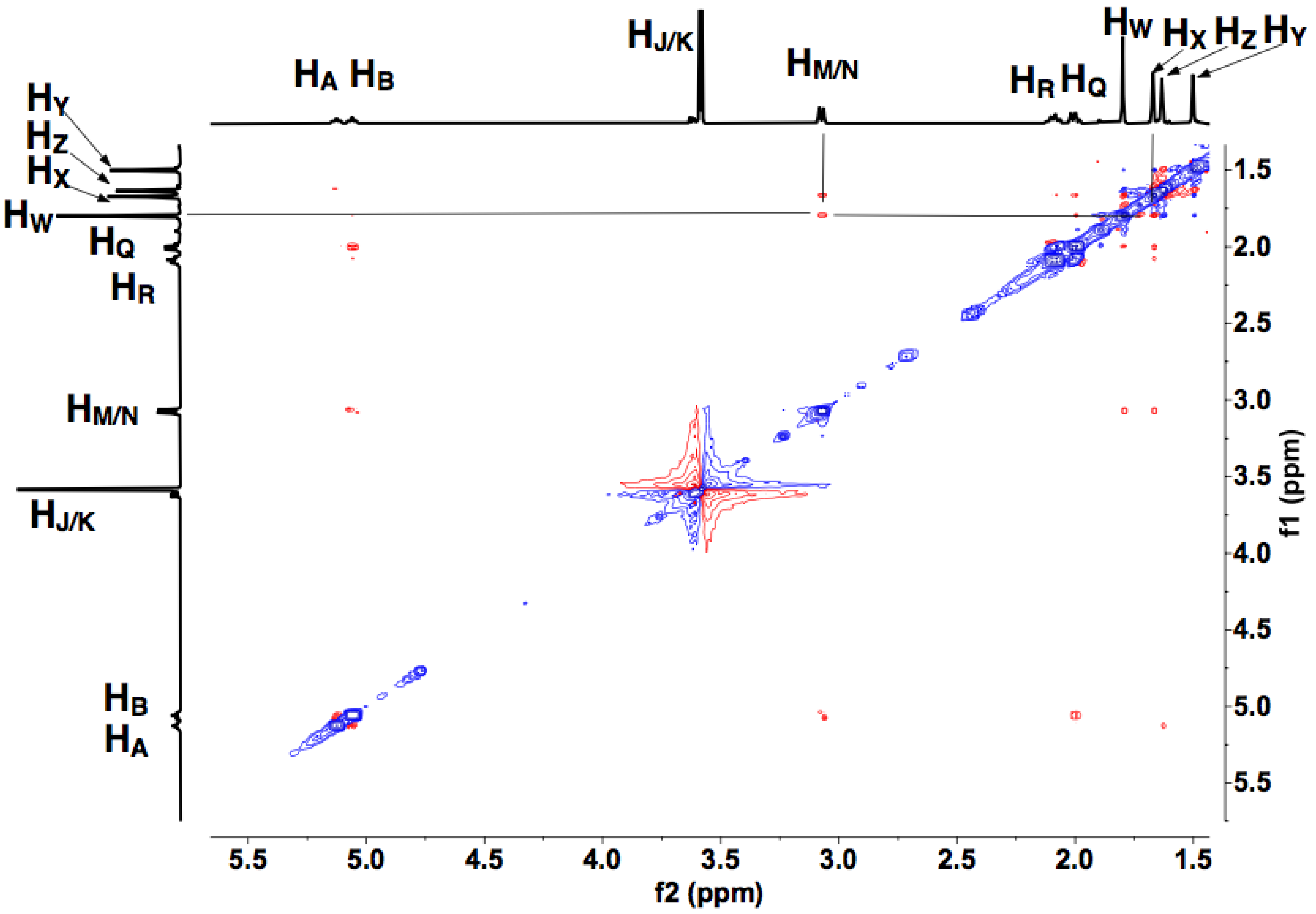

Fig. S20. ^{1}H-^{1}H 2D ROESY NMR (400 MHz) spectrum of 20.0 mM UQ-2 at 25 °C in C_6D_6. A standard ROESYAD pulse sequence was used consisting of 256 transients with 16 scans in the f1 domain using a 400 ms mixing time and 2.0 sec. relaxation delay per t1 increment.

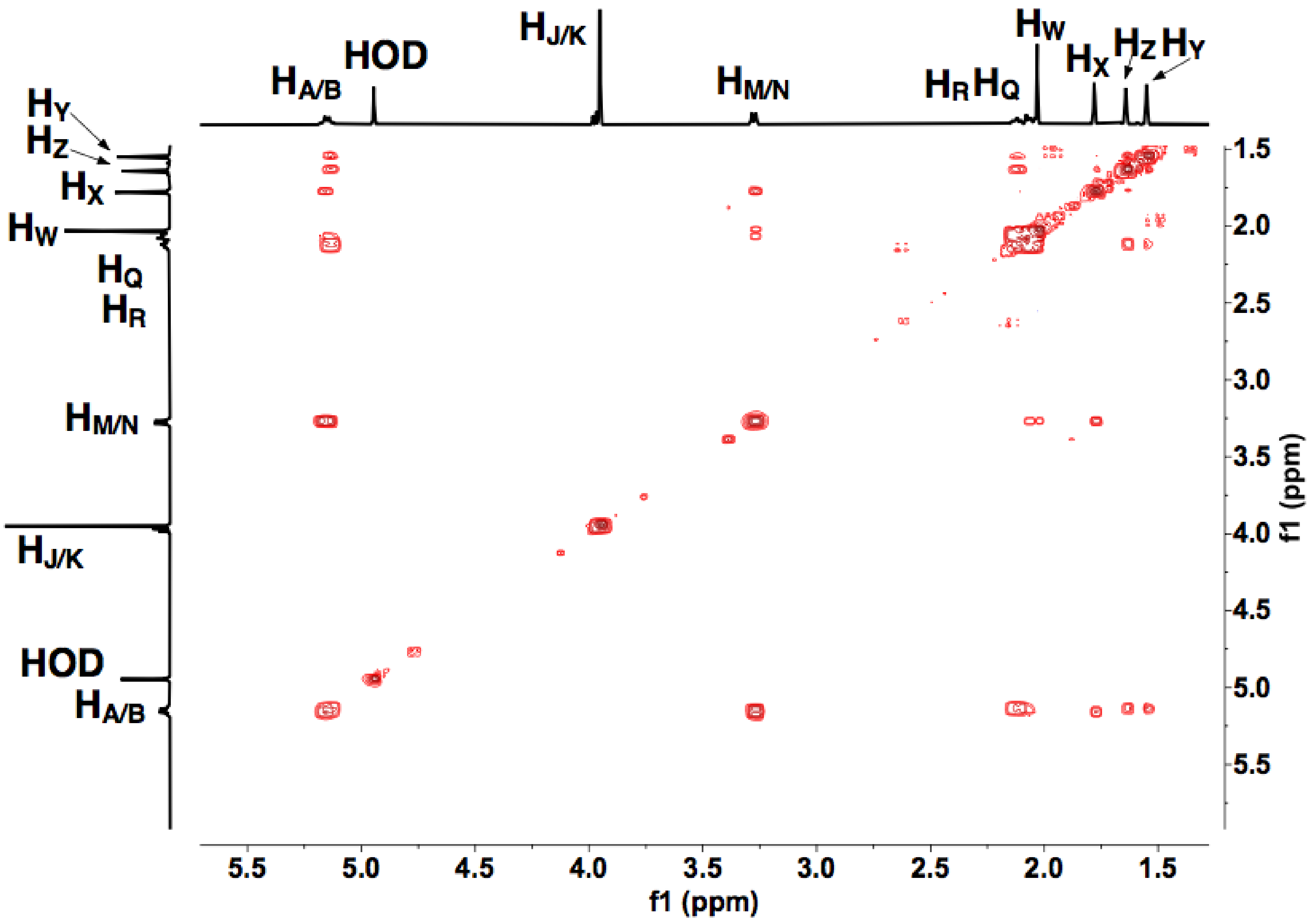

Fig. S21. ^1H-^1H 2D gCOSY NMR (400 MHz) spectrum of 20.0 mM UQ-2 in d_5-pyridine at 25 °C. A standard gCOSY pulse sequence was used consisting of 200 t1 increments with 8 scans per t1 increment.

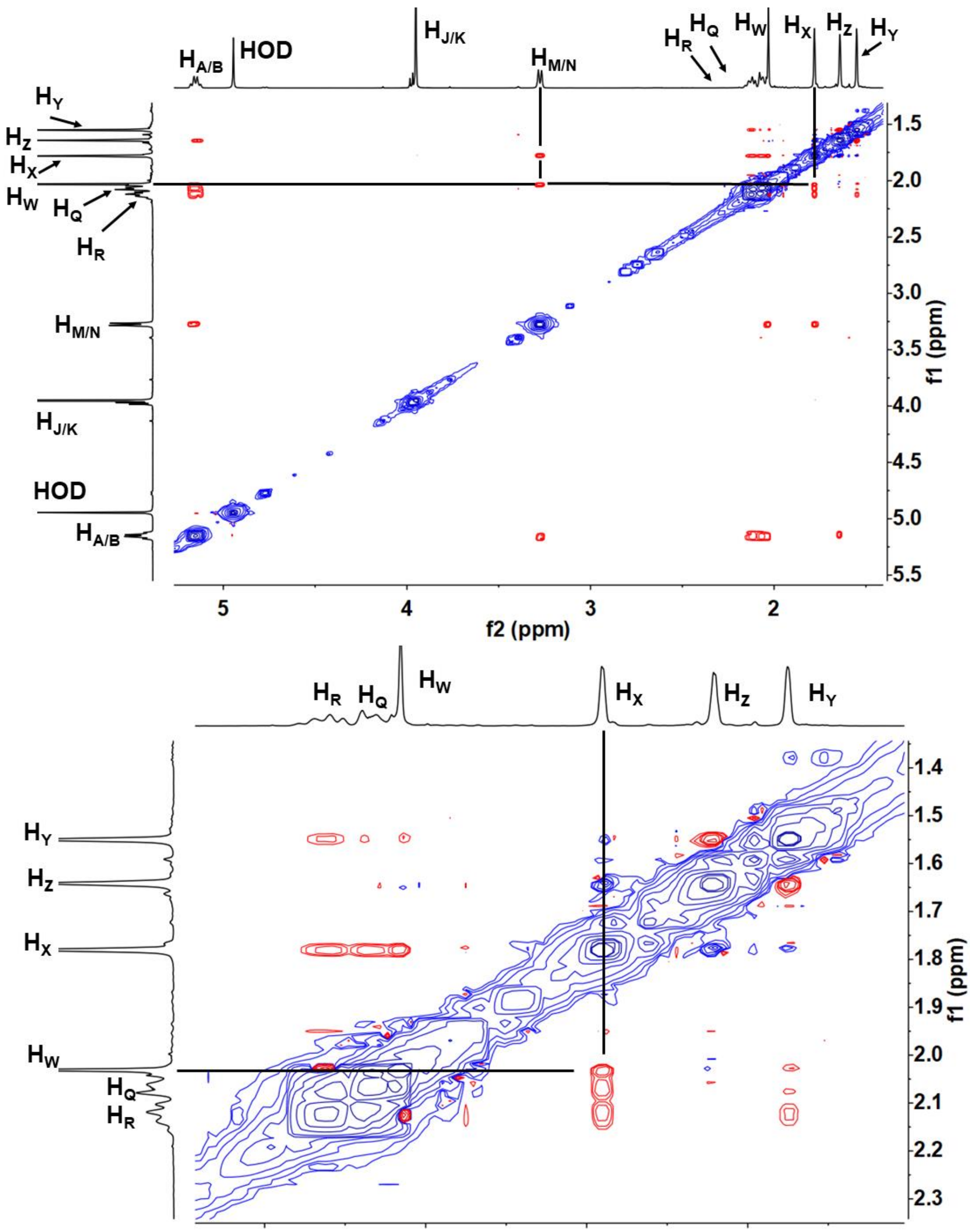

Figure S22. (A) ^{1}H-^{1}H 2D Full NOESY and **(B)** ^{1}H-^{1}H 2D Partial NOESY NMR (400 MHz) spectra of 20 mM UQ-2 at 25 °C in d_5-pyridine. A standard NOESY pulse sequence was used consisting of 256 transients with 16 scans in the f1 domain using a 500 ms mixing time and 1.5 sec relaxation delay.

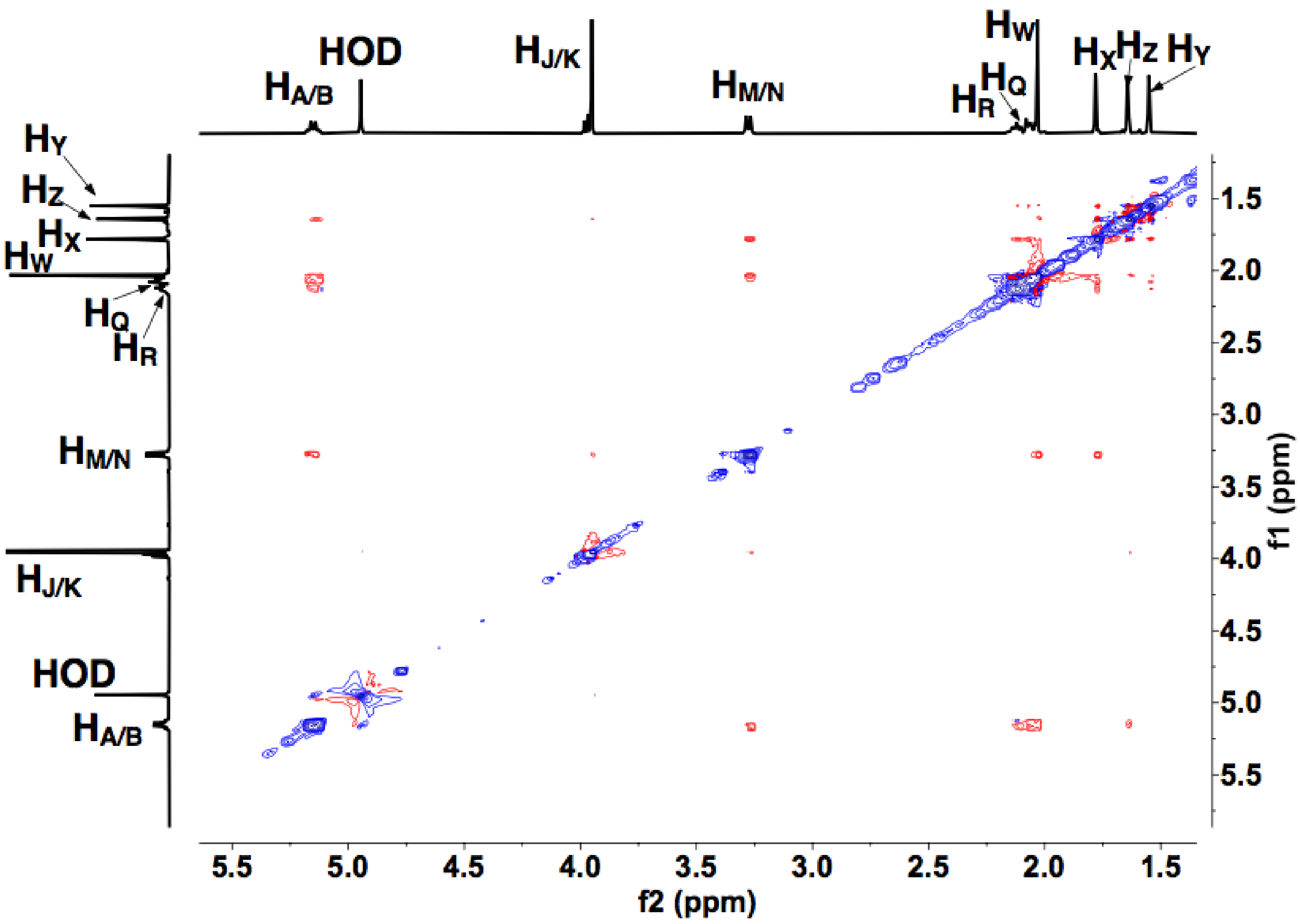

Fig. S23. ^1H-^1H 2D ROESY NMR (400 MHz) spectrum of 20.0 mM UQ-2 at 25 °C in d_5-pyridine. A standard ROESYAD pulse sequence was used consisting of 256 transients with 16 scans in the f1 domain using a 400 ms mixing time and 2.0 sec. relaxation delay per t1 increment.

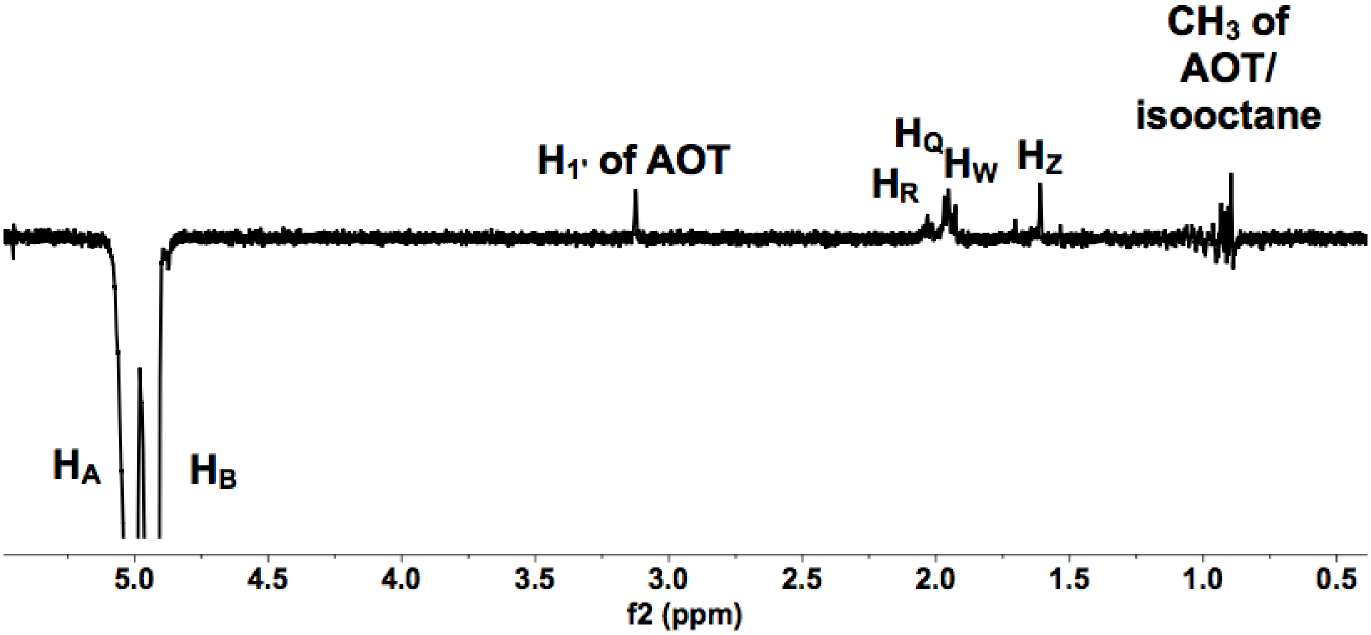

Figure S24. ^{1}H-^{1}H 1D NOESY in w_0 12 reverse micelles with irradiated H_A and H_B protons (5.14 ppm, 80.871 Hz). A NOESY1D pulse sequence consisted of 128 transients in the f1 domain using a 200 ms mixing time and 2.0 sec relaxation delay.

III Molecular Mechanics: conformational analysis of UQ-2 and MK-2 and proton distances

To generate visual aids for UQ-2 conformations, Merck Molecular Force Field 94 (MMFF94) Molecular Mechanics gas phase simulations were conducted using ChemBio3D Ultra 12.0 at 25 °C. Conformations were generated using ChemBio3D Ultra 12.0 where UQ-2 structures were built and then desired bonds rotated to reflect the inter-atomic distances observed by NMR spectroscopy. Conformations were then energy minimized with a root mean square (RMS) gradient of 0.1 and 10 iterations to obtain proper bond lengths and angles to result in the conformations that agreed with our interpretation of the cross-peak observations in the ^{1}H-^{1}H 2D NOESY and ROESY NMR spectral data.

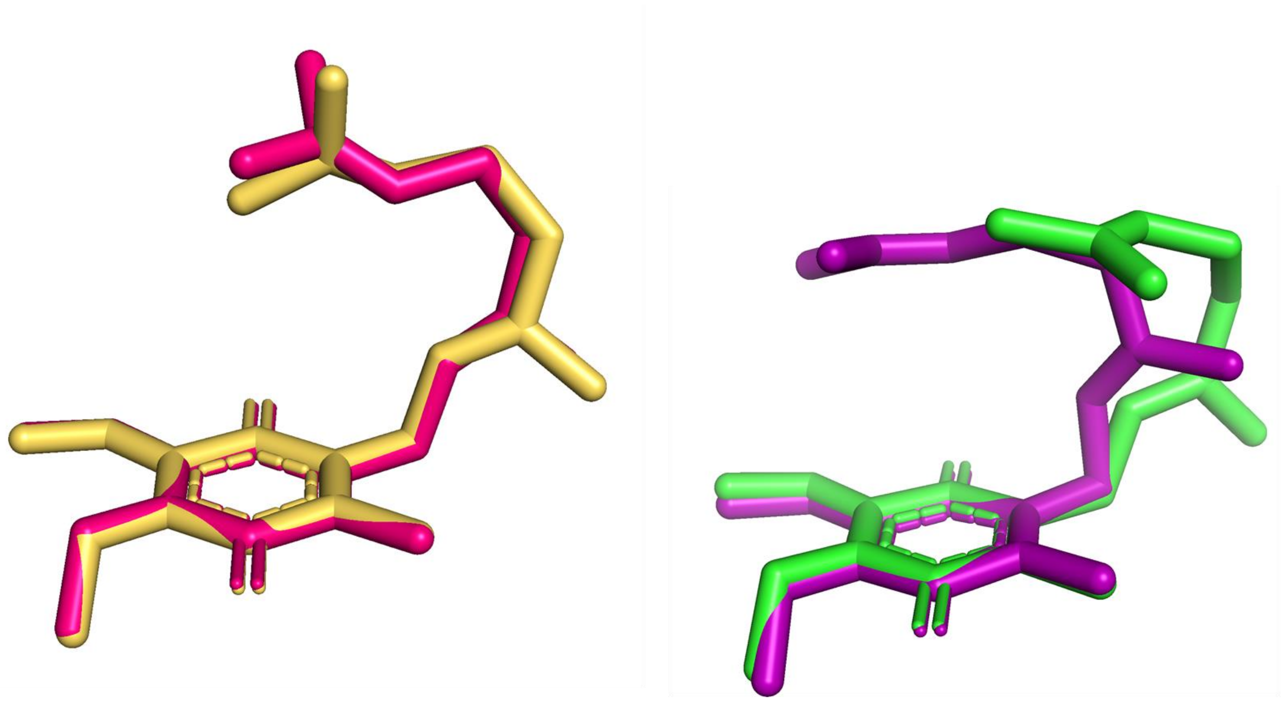

UQ-2 in benzene (yellow)
UQ-2 in pyridine (pink)

UQ-2 in acetonitrile (green)
UQ-2 in DMSO (purple)

Fig. S25 Comparison of superimposed 3D conformations of UQ-2 based on the 2D NMR data in d_3-acetonitrile / d_6-DMSO and d_6-benzene / d_5-pyridine. The images were generated using PyMOL molecular visualization system.

Table S1. Comparison of interproton distances of UQ-2 and MK-2 in d_6-DMSO and d_5-pyridine.

Proton Pair	d_6-DMSO		d_5-pyridine	
	UQ-2	MK-2	UQ-2	MK-2
$H_W – H_M$	3.6 Å	3.7 Å	3.7 Å	3.5 Å
$H_W – H_N$	2.1 Å	2.2 Å	2.3 Å	2.0 Å
$H_W – H_X$	3.2 Å	2.5 Å	3.9 Å	2.7 Å
$H_W – H_Y$	3.1 Å	2.6 Å	8.1 Å	6.1 Å
$H_W – H_Z$	5.1 Å	4.8 Å	5.3 Å	7.0 Å
$H_W – H_A$	6.2 Å	6.5 Å	3.9 Å	7.1 Å
H_W - H_B	5.0 Å	4.1 Å	2.9 Å	4.7 Å
$H_W – H_R$	4.7 Å	5.9 Å	5.5 Å	6.6 Å
H_W - H_Q	5.9 Å	4.7 Å	5.0 Å	5.4 Å

Table S2. Comparison of interproton distances of UQ-2 and MK-2 w_0 12 reverse micelles. The interproton distances for UQ-2 were measured from nearest proton to nearest proton (i.e. closest H_W proton to closest H_Y proton)

Proton Pair	UQ-2	MK-2
$H_W – H_M$	3.6 Å	3.7 Å
$H_W – H_N$	2.1 Å	2.2 Å
$H_W – H_X$	2.6 Å	2.7 Å
$H_W – H_Y$	4.6 Å	4.2 Å
$H_W – H_Z$	3.7 Å	4.0 Å
$H_W – H_A$	4.3 Å	5.6 Å
H_W - H_B	4.2 Å	4.1 Å
$H_W – H_R$	5.3 Å	5.6 Å
H_W - H_Q	4.9 Å	4.6 Å

IV Langmuir Monolayers for UQ-2

Methods: Compression Modulus Analysis of Langmuir Monolayers

The compression moduli of normalized compression isotherms were calculated with equation S1, where C_s^{-1} is the compression modulus, A is the normalized area per phospholipid (Å^2), and π is the surface pressure (mN/m).

$$C_s^{-1} = -A\left(\frac{d\pi}{dA}\right) \quad \text{(S1)}$$

Compression modulus calculations were performed in Origin 2021. The derivative of surface pressure was smoothed with a Savitsky-Golay function (2nd degree polynomial, 350 points per window) before being multiplied with negative area.

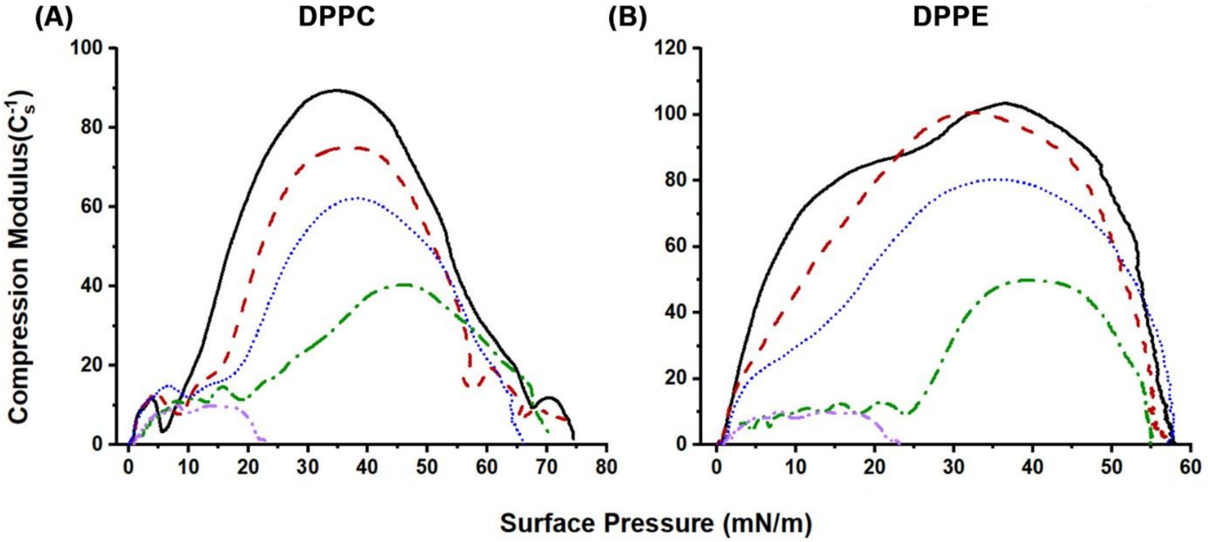

Figure S26. The compression moduli of normalized UQ-2 mixed monolayers of (A) DPPC or (B) DPPE. Solid black curves represent lipid controls, red dashed curves represent 75:25 lipid:UQ-2 films, blue dotted curves represent 50:50 lipid:UQ-2 films, green dash-dot curves represent 25:75 lipid:UQ-2 films, and purple dash-dot-dot curves represent UQ-2 films.

Table S3. Percent difference between the area of mixed DPPC:UQ-2 monolayers and the DPPC control. Positive numbers indicate an expansion of area while negative numbers suggest a condensation. The shaded rows indicate physiological surface pressure.

**Surface Pressure (mN/m)**	**75:25 DPPC:UQ-2**	**50:50 DPPC:UQ-2**	**25:75 DPPC:UQ-2**
**5**	10.9	26.6	92.9
**10**	19.5	49.2	111.9
**15**	14.0	39.0	91.0
**20**	8.6	28.0	66.8

**25**	6.8	21.6	48.4
**30**	6.2	19.0	36.5
**35**	5.6	17.4	27.7
**40**	5.5	16.6	22.3
**35**	4.5	15.4	18.5
**50**	4.0	15.0	15.7
**55**	4.6	13.5	14.1

Table S4. Percent difference between the area of mixed DPPE:UQ-2 monolayers and the DPPE control. Positive numbers indicate an expansion of area while negative numbers suggest a condensation. The shaded rows indicate physiological surface pressure.

**Surface Pressure (mN/m)**	**75:25 DPPE:UQ-2**	**50:50 DPPE:UQ-2**	**25:75 DPPE:UQ-2**
**5**	14.9	39.0	208.3
**10**	11.9	31.5	145.8
**15**	10.2	25.9	104.9
**20**	9.4	22.0	71.5
**25**	9.4	20.3	44.1
**30**	9.5	19.3	29.1
**35**	9.9	19.0	25.0
**40**	10.1	18.5	22.5
**35**	9.8	17.5	20.6
**50**	8.7	16.7	16.9
**55**	7.0	16.6	5.4

V Dynamic Light Scattering data for UQ-2

Table S5. Dynamic Light Scattering measurements on UQ-2 in 0.1 M AOT/isooctane RMs

w_0	N (probes)	N (Micelles)	N (probes) / N (Micelles)	(UBQ-2) r_h^a (nm)	(UBQ-2) PDI	Blank r_h^a (nm)	r_h^b Literature (nm)	PDI Empty RM
20	1.07 X 10^{18}	8.20 X 10^{17}	1.30	4.2 (± 0.1)	0.60 (± 0.14)	4.5 (± 0.3)	4.4	0.38 (± 0.28)
12	6.34 X 10^{17}	2.09 X 10^{18}	0.30	3.7 (± 0.6)	0.14 (± 0.04)	3.72 (± 0.02)	3.7	0.19 (± 0.01)
4	2.00 X 10^{17}	8.31 X 10^{19}	0.02	2.3 (± 0.3)	0.16 (± 0.03)	2.0 (± 0.1)	2.5	0.31 (± 0.05)

[a]Radius measurements were taken from the volume distribution
[b] Ref. 2.

RM Sample Preparation for Dynamic Light Scattering (DLS) Studies. RMs for DLS studies were prepared as described for the NMR spectroscopic studies except that distilled deionized (DDI) H_2O was used as the water pool instead of D_2O and the 0.50 M AOT was diluted with isooctane after the RM had formed to a final concentration of 0.1 M AOT.

DLS Measurements. The hydrodynamic radius of the RMs was determined by DLS measurements performed on a Malvern Zetasizer Nano ZS instrument (Malvern Instruments, Malvern, UK). The DLS cuvette (1 cm X 1 cm, glass) was washed out three times with isooctane followed by three washes with the RM sample. Then, the cuvette was filled with 1 mL of the RM sample and closed with a Teflon cap. Each experiment was conducted at 25 °C and consisted of a 700 sec. sample equilibration period followed by 10 measurements consisting of 15 scans each.[1] Each sample was measured in triplicate and the radius and pdi were recorded. The data were analyzed using Malvern Zetasizer Software Version 711 and compared to values reported in literature.[2]

DLS measurements of UQ-2 containing AOT/isooctane RMs. DLS established the formation of RMs. Samples of UQ-2 RMs were prepared using 0.10 M AOT/isooctane and the results are shown in Table S5. The average radius obtained from w_0 sizes 4, 12, and 20 compared favourably with those reported previously in the literature.[2, 3, 4] These results were observed with RMs prepared with and without UQ-2. These results showed that RMs formed, and that the presence of UQ-2 did not significantly affect the size or stability of the RM.

(1) Peters, B. J.; Groninger, A. S.; Fontes, F. L.; Crick, D. C.; Crans, D. C. *Langmuir* **2016**, *32*, 9451.
(2) Maitra, A. *J. Phys. Chem.* **1984**, *88*, 5122.
(3) Eicke, H. F.; Rehak, J. *Helv. Chim. Acta* **1976**, *59*, 2883.
(4) Zulauf, M.; Eicke, H. F. *J. Phys. Chem.* **1979**, *83*, 480.
